# Novel genetic loci associated with hippocampal volume

**DOI:** 10.1038/ncomms13624

**Published:** 2017-01-18

**Authors:** Derrek P. Hibar, Hieab H. H. Adams, Neda Jahanshad, Ganesh Chauhan, Jason L. Stein, Edith Hofer, Miguel E. Renteria, Joshua C. Bis, Alejandro Arias-Vasquez, M. Kamran Ikram, Sylvane Desrivières, Meike W. Vernooij, Lucija Abramovic, Saud Alhusaini, Najaf Amin, Micael Andersson, Konstantinos Arfanakis, Benjamin S. Aribisala, Nicola J. Armstrong, Lavinia Athanasiu, Tomas Axelsson, Ashley H. Beecham, Alexa Beiser, Manon Bernard, Susan H. Blanton, Marc M. Bohlken, Marco P. Boks, Janita Bralten, Adam M. Brickman, Owen Carmichael, M. Mallar Chakravarty, Qiang Chen, Christopher R. K. Ching, Vincent Chouraki, Gabriel Cuellar-Partida, Fabrice Crivello, Anouk Den Braber, Nhat Trung Doan, Stefan Ehrlich, Sudheer Giddaluru, Aaron L. Goldman, Rebecca F. Gottesman, Oliver Grimm, Michael E. Griswold, Tulio Guadalupe, Boris A. Gutman, Johanna Hass, Unn K. Haukvik, David Hoehn, Avram J. Holmes, Martine Hoogman, Deborah Janowitz, Tianye Jia, Kjetil N. Jørgensen, Nazanin Karbalai, Dalia Kasperaviciute, Sungeun Kim, Marieke Klein, Bernd Kraemer, Phil H. Lee, David C. M. Liewald, Lorna M. Lopez, Michelle Luciano, Christine Macare, Andre F. Marquand, Mar Matarin, Karen A. Mather, Manuel Mattheisen, David R. McKay, Yuri Milaneschi, Susana Muñoz Maniega, Kwangsik Nho, Allison C. Nugent, Paul Nyquist, Loes M. Olde Loohuis, Jaap Oosterlaan, Martina Papmeyer, Lukas Pirpamer, Benno Pütz, Adaikalavan Ramasamy, Jennifer S. Richards, Shannon L. Risacher, Roberto Roiz-Santiañez, Nanda Rommelse, Stefan Ropele, Emma J. Rose, Natalie A. Royle, Tatjana Rundek, Philipp G. Sämann, Arvin Saremi, Claudia L. Satizabal, Lianne Schmaal, Andrew J. Schork, Li Shen, Jean Shin, Elena Shumskaya, Albert V. Smith, Emma Sprooten, Lachlan T. Strike, Alexander Teumer, Diana Tordesillas-Gutierrez, Roberto Toro, Daniah Trabzuni, Stella Trompet, Dhananjay Vaidya, Jeroen Van der Grond, Sven J. Van der Lee, Dennis Van der Meer, Marjolein M. J. Van Donkelaar, Kristel R. Van Eijk, Theo G. M. Van Erp, Daan Van Rooij, Esther Walton, Lars T. Westlye, Christopher D. Whelan, Beverly G. Windham, Anderson M. Winkler, Katharina Wittfeld, Girma Woldehawariat, Christiane Wolf, Thomas Wolfers, Lisa R. Yanek, Jingyun Yang, Alex Zijdenbos, Marcel P. Zwiers, Ingrid Agartz, Laura Almasy, David Ames, Philippe Amouyel, Ole A. Andreassen, Sampath Arepalli, Amelia A. Assareh, Sandra Barral, Mark E. Bastin, Diane M. Becker, James T. Becker, David A. Bennett, John Blangero, Hans van Bokhoven, Dorret I. Boomsma, Henry Brodaty, Rachel M. Brouwer, Han G. Brunner, Randy L. Buckner, Jan K. Buitelaar, Kazima B. Bulayeva, Wiepke Cahn, Vince D. Calhoun, Dara M. Cannon, Gianpiero L. Cavalleri, Ching-Yu Cheng, Sven Cichon, Mark R. Cookson, Aiden Corvin, Benedicto Crespo-Facorro, Joanne E. Curran, Michael Czisch, Anders M. Dale, Gareth E. Davies, Anton J. M. De Craen, Eco J. C. De Geus, Philip L. De Jager, Greig I. De Zubicaray, Ian J. Deary, Stéphanie Debette, Charles DeCarli, Norman Delanty, Chantal Depondt, Anita DeStefano, Allissa Dillman, Srdjan Djurovic, Gary Donohoe, Wayne C. Drevets, Ravi Duggirala, Thomas D. Dyer, Christian Enzinger, Susanne Erk, Thomas Espeseth, Iryna O. Fedko, Guillén Fernández, Luigi Ferrucci, Simon E. Fisher, Debra A. Fleischman, Ian Ford, Myriam Fornage, Tatiana M. Foroud, Peter T. Fox, Clyde Francks, Masaki Fukunaga, J. Raphael Gibbs, David C. Glahn, Randy L. Gollub, Harald H. H. Göring, Robert C. Green, Oliver Gruber, Vilmundur Gudnason, Sebastian Guelfi, Asta K. Håberg, Narelle K. Hansell, John Hardy, Catharina A. Hartman, Ryota Hashimoto, Katrin Hegenscheid, Andreas Heinz, Stephanie Le Hellard, Dena G. Hernandez, Dirk J. Heslenfeld, Beng-Choon Ho, Pieter J. Hoekstra, Wolfgang Hoffmann, Albert Hofman, Florian Holsboer, Georg Homuth, Norbert Hosten, Jouke-Jan Hottenga, Matthew Huentelman, Hilleke E. Hulshoff Pol, Masashi Ikeda, Clifford R. Jack Jr, Mark Jenkinson, Robert Johnson, Erik G. Jönsson, J. Wouter Jukema, René S. Kahn, Ryota Kanai, Iwona Kloszewska, David S. Knopman, Peter Kochunov, John B. Kwok, Stephen M. Lawrie, Hervé Lemaître, Xinmin Liu, Dan L. Longo, Oscar L. Lopez, Simon Lovestone, Oliver Martinez, Jean-Luc Martinot, Venkata S. Mattay, Colm McDonald, Andrew M. McIntosh, Francis J. McMahon, Katie L. McMahon, Patrizia Mecocci, Ingrid Melle, Andreas Meyer-Lindenberg, Sebastian Mohnke, Grant W. Montgomery, Derek W. Morris, Thomas H. Mosley, Thomas W. Mühleisen, Bertram Müller-Myhsok, Michael A. Nalls, Matthias Nauck, Thomas E. Nichols, Wiro J. Niessen, Markus M. Nöthen, Lars Nyberg, Kazutaka Ohi, Rene L. Olvera, Roel A. Ophoff, Massimo Pandolfo, Tomas Paus, Zdenka Pausova, Brenda W. J. H. Penninx, G. Bruce Pike, Steven G. Potkin, Bruce M. Psaty, Simone Reppermund, Marcella Rietschel, Joshua L. Roffman, Nina Romanczuk-Seiferth, Jerome I. Rotter, Mina Ryten, Ralph L. Sacco, Perminder S. Sachdev, Andrew J. Saykin, Reinhold Schmidt, Helena Schmidt, Peter R. Schofield, Sigurdur Sigursson, Andrew Simmons, Andrew Singleton, Sanjay M. Sisodiya, Colin Smith, Jordan W. Smoller, Hilkka Soininen, Vidar M. Steen, David J. Stott, Jessika E. Sussmann, Anbupalam Thalamuthu, Arthur W. Toga, Bryan J. Traynor, Juan Troncoso, Magda Tsolaki, Christophe Tzourio, Andre G. Uitterlinden, Maria C. Valdés Hernández, Marcel Van der Brug, Aad van der Lugt, Nic J. A. van der Wee, Neeltje E. M. Van Haren, Dennis van 't Ent, Marie-Jose Van Tol, Badri N. Vardarajan, Bruno Vellas, Dick J. Veltman, Henry Völzke, Henrik Walter, Joanna M. Wardlaw, Thomas H. Wassink, Michael E. Weale, Daniel R. Weinberger, Michael W. Weiner, Wei Wen, Eric Westman, Tonya White, Tien Y. Wong, Clinton B. Wright, Ronald H. Zielke, Alan B. Zonderman, Nicholas G. Martin, Cornelia M. Van Duijn, Margaret J. Wright, W. T. Longstreth, Gunter Schumann, Hans J. Grabe, Barbara Franke, Lenore J. Launer, Sarah E. Medland, Sudha Seshadri, Paul M. Thompson, M. Arfan Ikram

**Affiliations:** 1 Imaging Genetics Center, USC Mark and Mary Stevens Neuroimaging & Informatics Institute, Keck School of Medicine of University of Southern California, Los Angeles, California 90292, USA; 2 Department of Epidemiology, Erasmus University Medical Center, 3015 CE Rotterdam, The Netherlands; 3 Department of Radiology and Nuclear Medicine, Erasmus MC, 3015 CE Rotterdam, The Netherlands; 4 INSERM Unit U1219, University of Bordeaux, 33076 Bordeaux, France; 5 Department of Genetics & UNC Neuroscience Center, University of North Carolina (UNC), Chapel Hill, North Carolina, 27599, USA; 6 Department of Neurology, Clinical Division of Neurogeriatrics, Medical University Graz, Auenbruggerplatz 22, 8036 Graz, Austria; 7 Institute of Medical Informatics, Statistics and Documentation, Medical University Graz, Auenbruggerplatz 22, 8036 Graz, Austria; 8 QIMR Berghofer Medical Research Institute, Brisbane, Queensland 4006, Australia; 9 Cardiovascular Health Research Unit, Department of Medicine, University of Washington, 1730 Minor Avenue/Suite 1360. Seattle, Washington 98101, USA; 10 Department of Human Genetics, Radboud University Medical Center, 6525 GA Nijmegen, The Netherlands; 11 Department of Psychiatry, Radboud University Medical Center, 6525 GA Nijmegen, The Netherlands; 12 Department of Cognitive Neuroscience, Radboud University Medical Center, 6525 GA Nijmegen, The Netherlands; 13 Donders Institute for Brain, Cognition and Behaviour, Radboud University, 6525 GA Nijmegen, The Netherlands; 14 Academic Medicine Research Institute, Duke-NUS Graduate Medical School, Singapore, 169857, Singapore; 15 Singapore Eye Research Institute, Singapore National Eye Centre, Singapore, 168751, Singapore; 16 Memory Aging & Cognition Centre (MACC), National University Health System, Singapore, 119228, Singapore; 17 Department of Pharmacology, National University of Singapore, Singapore, 119077, Singapore; 18 MRC-SGDP Centre, Institute of Psychiatry, Psychology and Neuroscience, King's College London, London SE5 8AF, UK; 19 Brain Center Rudolf Magnus, Department of Psychiatry, UMC Utrecht, 3584 CX Utrecht, The Netherlands; 20 Department of Neurology and Neurosurgery, Montreal Neurological Institute, McGill University, Montreal, Quebec, Canada H3A 2B4; 21 The Royal College of Surgeons in Ireland, 123 St Stephen's Green, Dublin 2, Ireland; 22 Department of Integrative Medical Biology and Umeå Center for Functional Brain Imaging, Umeå University, 901 87 Umeå, Sweden; 23 Department of Biomedical Engineering, Illinois Institute of Technology, Chicago, Illinois 60616, USA; 24 Rush Alzheimer's Disease Center, Rush University Medical Center, Chicago, Illinois 60612, USA; 25 Department of Diagnostic Radiology and Nuclear Medicine, Rush University Medical Center, Chicago, Illinois 60616, USA; 26 Brain Research Imaging Centre, University of Edinburgh, Edinburgh EH4 2XU, UK; 27 Department of Computer Science, Lagos State University, Lagos, P.M.B. 01 LASU, Nigeria; 28 Scottish Imaging Network, A Platform for Scientific Excellence (SINAPSE) Collaboration, Department of Neuroimaging Sciences, University of Edinburgh, Edinburgh EH16 4SB, UK; 29 Centre for Healthy Brain Ageing, School of Psychiatry, University of New South Wales, Sydney, New South Wales 2052, Australia; 30 Mathematics and Statistics, Murdoch University, Perth, Western Australia, 6150, Australia; 31 NORMENT—KG Jebsen Centre, Institute of Clinical Medicine, University of Oslo, 0315 Oslo, Norway; 32 NORMENT—KG Jebsen Centre, Division of Mental Health and Addiction, Oslo University Hospital, 0424 Oslo, Norway; 33 Department of Medical Sciences, Molecular Medicine and Science for Life Laboratory, Uppsala University, Box 1432, SE-751 44 Uppsala, Sweden; 34 Dr John T. Macdonald Foundation Department of Human Genetics, University of Miami, Miller School of Medicine, Miami, Florida, 33136, USA; 35 John P. Hussman Institute for Human Genomics, University of Miami, Miller School of Medicine, Miami, Florida, 33136, USA; 36 Department of Neurology, Boston University School of Medicine, Boston, Massachusetts,02118, USA; 37 Department of Biostatistics, Boston University School of Public Health, Boston, Massachusetts 02118 USA; 38 Framingham Heart Study, 17 Mount Wayte Avenue, Framingham, Massachusetts 01703 USA; 39 Hospital for Sick Children, University of Toronto, Toronto, Ontario, Canada M5G 1X8; 40 Taub Institute for Research on Alzheimer's Disease and the Aging Brain; G.H. Sergievsky Center; Department of Neurology. Columbia University Medical Center, 639 West 1168th Street, New York, New York 10032, USA; 41 Pennington Biomedical Research Center, Baton Rouge, Louisiana 70808, USA; 42 Cerebral Imaging Centre, Douglas Mental Health University Institute, Montreal, Quebec, Canada H4H 1R3; 43 Department of Psychiatry and Biomedical Engineering, McGill University, Montreal, Quebec, Canada H3A 2B4; 44 Lieber Institute for Brain Development, Baltimore, Maryland 21205, USA; 45 Interdepartmental Neuroscience Graduate Program, UCLA School of Medicine, Los Angeles, California 90095, USA; 46 Lille University, Inserm, CHU Lille, Institut Pasteur de Lille, U1167—RID-AGE—Risk factors and molecular determinants of aging-related diseases, F-59000 Lille, France; 47 IMN UMR5293, GIN, CNRS, CEA, University of Bordeaux, 146 rue Léo Saignat, 33076 Bordeaux, France; 48 Biological Psychology, Amsterdam Neuroscience, Vrije Universiteit & Vrije Universiteit Medical Center, 1081 BT Amsterdam, The Netherlands; 49 Division of Psychological and Social Medicine and Developmental Neurosciences, Faculty of Medicine, TU Dresden, 01307 Dresden, Germany; 50 Department of Psychiatry, Massachusetts General Hospital, Boston, Massachusetts 02114, USA; 51 Martinos Center for Biomedical Imaging, Massachusetts General Hospital, Charlestown, Massachusetts 02129, USA; 52 NORMENT—KG Jebsen Centre for Psychosis Research, Department of Clinical Science, University of Bergen, 5021 Bergen, Norway; 53 Dr Einar Martens Research Group for Biological Psychiatry, Center for Medical Genetics and Molecular Medicine, Haukeland University Hospital, 5021 Bergen, Norway; 54 Department of Neurology, Johns Hopkins University School of Medicine, Baltimore, Maryland 21287, USA; 55 Central Institute of Mental Health, Medical Faculty Mannheim, University Heidelberg, 68159 Mannheim, Germany; 56 Department of Data Science, University of Mississippi Medical Center, Jackson, Mississippi, 39216, USA; 57 Language and Genetics Department, Max Planck Institute for Psycholinguistics, 6525 XD Nijmegen, The Netherlands; 58 International Max Planck Research School for Language Sciences, 6525 XD Nijmegen, The Netherlands; 59 Department of Child and Adolescent Psychiatry, Faculty of Medicine of the TU Dresden, 01307 Dresden, Germany; 60 Department of Research and Development, Diakonhjemmet Hospital, 0319 Oslo, Norway; 61 Max Planck Institute of Psychiatry, 80804 Munich, Germany; 62 Department of Psychology, Yale University, New Haven, Connecticut 06520, USA; 63 Department of Psychiatry, University Medicine Greifswald, 17489 Greifswald, Germany; 64 UCL Institute of Neurology, London, United Kingdom and Epilepsy Society, Bucks, SL9 0RJ, UK; 65 Department of Medicine, Imperial College London, London SW7 2AZ, UK; 66 Center for Neuroimaging, Radiology and Imaging Sciences, Indiana University School of Medicine, Indianapolis, Indiana 46202, USA; 67 Center for Computational Biology and Bioinformatics, Indiana University School of Medicine, Indianapolis, Indiana 46202, USA; 68 Indiana Alzheimer Disease Center, Indiana University School of Medicine, Indianapolis, Indiana 46202, USA; 69 Section for Experimental Psychopathology and Neuroimaging, Department of General Psychiatry, Heidelberg University, Heidelberg, 69120, Germany; 70 Psychiatric and Neurodevelopmental Genetics Unit, Center for Human Genetic Research, Massachusetts General Hospital, Boston, Massachusetts 02114, USA; 71 Harvard Medical School, Boston, Massachusetts 02115, USA; 72 Stanley Center for Psychiatric Research, Broad Institute of MIT and Harvard, Boston, Massachusetts 02141, USA; 73 Lurie Center for Autism, Massachusetts General Hospital, Harvard Medical School, Lexington, Massachusetts, 02421, USA; 74 Centre for Cognitive Ageing and Cognitive Epidemiology, Psychology, University of Edinburgh, Edinburgh EH8 9JZ, UK; 75 Donders Centre for Cognitive Neuroimaging, Radboud University, Nijmegen, 6525 EN, The Netherlands; 76 Reta Lila Weston Institute and Department of Molecular Neuroscience, UCL Institute of Neurology, London WC1N 3BG, UK; 77 Department of Biomedicine, Aarhus University, DK-8000 Aarhus, Denmark; 78 The Lundbeck Foundation Initiative for Integrative Psychiatric Research, iPSYCH, DK-8000 Aarhus and Copenhagen, Denmark; 79 Center for integrated Sequencing, iSEQ, Aarhus University, DK-8000 Aarhus, Denmark; 80 Department of Psychiatry, Yale University, New Haven, Connecticut 06511, USA; 81 Olin Neuropsychiatric Research Center, Hartford, Connecticut 06114, USA; 82 Department of Psychiatry, EMGO Institute for Health and Care Research and Neuroscience Campus Amsterdam, VU University Medical Center/GGZ inGeest, 1081 HL Amsterdam, The Netherlands; 83 Human Genetics Branch, National Institute of Mental Health Intramural Research Program, 35 Convent Drive, Rm 1A202, Bethesda, Maryland 20892-3719, USA; 84 Department of Neurology, Department of Anesthesia/Critical Care Medicine, Department of Neurosurgery, Johns Hopkins, USA600 N. Wolfe St, Baltimore, Maryland 21287, USA; 85 Center for Neurobehavioral Genetics, University of California, Los Angeles, California 90095, USA; 86 Department of Clinical Neuropsychology, VU University Amsterdam, Amsterdam, 1081 HV, The Netherlands; 87 Division of Psychiatry, Royal Edinburgh Hospital, University of Edinburgh, Edinburgh EH10 5HF, UK; 88 Division of Systems Neuroscience of Psychopathology, Translational Research Center, University Hospital of Psychiatry, University of Bern, Bern, 3060, Switzerland; 89 Department of Medical and Molecular Genetics, King's College London, London SE1 9RT, UK; 90 The Jenner Institute Laboratories, University of Oxford, Oxford OX3 7DQ, UK; 91 Karakter Child and Adolescent Psychiatry University Center, Nijmegen, 6525 GC, The Netherlands; 92 Department of Medicine and Psychiatry, University Hospital Marqués de Valdecilla, School of Medicine, University of Cantabria-IDIVAL, 39008 Santander, Spain; 93 CIBERSAM (Centro Investigación Biomédica en Red Salud Mental), Santander, 39011, Spain; 94 Psychosis Research Group, Department of Psychiatry & Trinity Translational Medicine Institute, Trinity College, Dublin, Dublin 2, Ireland; 95 Centre for Clinical Brain Sciences, University of Edinburgh, Edinburgh EH16 4SB, UK; 96 Department of Neurology, University of Miami, Miller School of Medicine, Miami, Florida, 33136, USA; 97 Department of Epidemiology and Public Health Sciences, University of Miami, Miller School of Medicine, Miami, Florida, 33136, USA; 98 Orygen, The National Centre of Excellence in Youth Mental Health, Melbourne, Victoria, 3502, Australia; 99 Centre for Youth Mental Health, The University of Melbourne, Melbourne, Victoria, 3502, Australia; 100 Department of Psychiatry, Neuroscience Campus Amsterdam, VU University Medical Center, 1007 MB Amsterdam, The Netherlands; 101 Multimodal Imaging Laboratory, Department of Neurosciences, University of California, San Diego, California 92093, USA; 102 Department of Cognitive Sciences, University of California, San Diego, California 92161, USA; 103 Icelandic Heart Association, Kopavogur, 201, Iceland; 104 Faculty of Medicine, University of Iceland, Reykjavik, 101, Iceland; 105 Department of Psychiatry, Icahn School of Medicine at Mount Sinai, New York, New York, 10029, USA; 106 Queensland Brain Institute, University of Queensland, Brisbane, Queensland 4072, Australia; 107 Institute for Community Medicine, University Medicine Greifswald, 17489 Greifswald, Germany; 108 Neuroimaging Unit, Technological Facilities. Valdecilla Biomedical Research Institute IDIVAL, Santander, Cantabria, 39011, Spain; 109 Institut Pasteur, 75015 Paris, France; 110 Department of Genetics, King Faisal Specialist Hospital and Research Centre, Riyadh 11211, Saudi Arabia; 111 Department of Cardiology, Leiden University Medical Center, Leiden, 2300RC, The Netherlands; 112 GeneSTAR Research Center, Department of Medicine, Johns Hopkins University School of Medicine, 1830 E Monument St Suite 8028, Baltimore, Maryland 21287, USA; 113 Department of Radiology, Leiden University Medical Center, Leiden, 2300RC, The Netherlands; 114 Department of Psychiatry, University of Groningen, University Medical Center Groningen, Groningen, 9700RB, The Netherlands; 115 Brain Center Rudolf Magnus, Human Neurogenetics Unit, UMC Utrecht, 3584 CG Utrecht, The Netherlands; 116 Department of Psychiatry and Human Behavior, University of California-Irvine, Irvine, California 92617, USA; 117 Department of Psychology, Georgia State University, Atlanta, Georgia 30302, USA; 118 NORMENT—KG Jebsen Centre, Department of Psychology, University of Oslo, 0317 Oslo, Norway; 119 Department of Medicine, University of Mississippi Medical Center, Jackson, Mississippi, 39216, USA; 120 FMRIB Centre, University of Oxford, Oxford OX3 9DU, UK; 121 German Center for Neurodegenerative Diseases (DZNE) Rostock/Greifswald, 17487 Greifswald, Germany; 122 University of Wuerzburg, Department of Psychiatry, Psychosomatics and Psychotherapy, Wuerzburg, 97080, Germany; 123 Department of Neurological Sciences, Rush University Medical Center, Chicago, Illinois 60612, USA; 124 Biospective Inc, Montreal, Quebec, Canada, 6100 Avenue Royalmount, Montréal, Québec, Canada H4P 2R2; 125 Department of Clinical Neuroscience, Centre for Psychiatric Research, Karolinska Institutet, SE-171 77 Stockholm, Sweden; 126 South Texas Diabetes and Obesity Institute, University of Texas Rio Grande Valley School of Medicine, Brownsville/Edinburg/San Antonio, Texas, 78250, USA; 127 Department of Genetics, Perelman School of Medicine, University of Pennsylvania, Philadelphia, Pennsylvania 19104, USA; 128 Department of Biomedical and Health Informatics, The Children's Hospital of Philadelphia, Philadelphia, Pennsylvania 29104, USA; 129 National Ageing Research Institute, Royal Melbourne Hospital, Melbourne, Victoria 3052, Australia; 130 Academic Unit for Psychiatry of Old Age, University of Melbourne, Melbourne, Victoria 3101, Australia; 131 Laboratory of Neurogenetics, National Institute on Aging, National Institutes of Health, Bethesda, Maryland 20892, USA; 132 Departments of Psychiatry, Neurology, and Psychology, University of Pittsburgh, 3501 Forbes Ave., Suite 830, Pittsburgh, Pennsylvania 15213, USA; 133 Dementia Collaborative Research Centre—Assessment and Better Care, University of New South Wales, Sydney, New South Wales 2052, Australia; 134 Department of Clinical Genetics and GROW School for Oncology and Developmental Biology, Maastricht University Medical Center, 6200 MD Maastricht, The Netherlands; 135 Department of Psychology, Center for Brain Science, Harvard University, Cambridge, Massachusetts 02138, USA; 136 Department of Evolution and Genetics, Dagestan State University, Makhachkala 367000, Dagestan, Russia; 137 The Mind Research Network & LBERI, Albuquerque, New Mexico 87106, USA; 138 Department of ECE, University of New Mexico, Albuquerque, New Mexico 87131, USA; 139 Centre for Neuroimaging & Cognitive Genomics (NICOG), Clinical Neuroimaging Laboratory, NCBES Galway Neuroscience Centre, College of Medicine Nursing and Health Sciences, National University of Ireland Galway, H91 TK33 Galway, Ireland; 140 Department of Ophthalmology, Yong Loo Lin School of Medicine, National University of Singapore, Singapore, 119077, Singapore; 141 Division of Medical Genetics, Department of Biomedicine, University of Basel, 4031 Basel, Switzerland; 142 Institute of Human Genetics, University of Bonn, 53127 Bonn, Germany; 143 Institute of Neuroscience and Medicine (INM-1), Research Centre Jülich, 52425 Jülich, Germany; 144 Center for Multimodal Imaging and Genetics, University of California, San Diego, California 92093, USA; 145 Departments of Neurosciences, Radiology, Psychiatry, and Cognitive Science, University of California, San Diego, California 92093, USA; 146 Avera Institute for Human Genetics, Sioux Falls, South Dakota 57108, USA; 147 Department of Gerontology and Geriatrics, Leiden University Medical Center, Leiden, 2300RC, The Netherlands; 148 Program in Translational NeuroPsychiatric Genomics, Departments of Neurology & Psychiatry, Brigham and Women's Hospital, Boston, Massachusetts, 02115, USA; 149 Harvard Medical School, Boston, Massachusetts, 02115, USA; 150 Program in Medical and Population Genetics, Broad Institute, Cambridge, Massachusetts, 02142, USA; 151 Broad Institute, Cambridge, Massachusetts, 02142, USA; 152 Faculty of Health and Institute of Health and Biomedical Innovation, Queensland University of Technology (QUT), Brisbane, Queensland 4059, Australia; 153 Department of Neurology, Bordeaux University Hospital, Bordeaux, 33076, France; 154 Imaging of Dementia and Aging (IDeA) Laboratory, Department of Neurology and Center for Neuroscience, University of California at Davis, 4860 Y Street, Suite 3700, Sacramento, California 95817, USA; 155 Neurology Division, Beaumont Hospital, Dublin 9, Ireland; 156 Department of Neurology, Hopital Erasme, Universite Libre de Bruxelles, 1070 Brussels, Belgium; 157 Department of Medical Genetics, Oslo University Hospital, 0420 Oslo, Norway; 158 Cognitive Genetics and Cognitive Therapy Group, Neuroimaging, Cognition & Genomics Centre (NICOG) & NCBES Galway Neuroscience Centre, School of Psychology and Discipline of Biochemistry, National University of Ireland Galway, H91 TK33, Galway, Ireland; 159 Neuropsychiatric Genetics Research Group, Department of Psychiatry and Trinity College Institute of Psychiatry, Trinity College Dublin, Dublin 8, Ireland; 160 Janssen Research & Development, LLC, Titusville, New Jersey 08560, USA; 161 Charité - Universitätsmedizin Berlin, Campus Charité Mitte, Department of Psychiatry and Psychotherapy, Charitéplatz 1, 10117 Berlin, Germany; 162 Intramural Research Program of the National Institute on Aging, Baltimore, Maryland, 21224, USA; 163 Department of Neurological Sciences & Department of Behavioral Sciences, Rush University Medical Center, Chicago, Illinois 60616, USA; 164 Robertson Centre for Biostatistics, University of Glasgow, Glasgow, G41 4DQ, UK; 165 Institute of Molecular Medicine and Human Genetics Center, University of Texas Health Science Center at Houston, Houston, Texas, 77030, USA; 166 Medical and Molecular Genetics, Indiana University School of Medicine, Indianapolis, Indiana 46202, USA; 167 University of Texas Health Science Center, San Antonio, Texas 78229, USA; 168 Division of Cerebral Integration, National Institute for Physiological Sciences, Aichi, 444-8585, Japan; 169 Division of Genetics, Department of Medicine, Brigham and Women's Hospital, Boston, Massachusetts 02115, USA; 170 Department of Neuroscience, Faculty of Medicine, Norwegian University of Science and Technology (NTNU), Trondheim, 7491, Norway; 171 Department of Radiology, St. Olav's Hospital, Trondheim University Hospital, Trondheim, 7030, Norway; 172 Department of Psychiatry, Osaka University Graduate School of Medicine, Osaka 565-0871, Japan; 173 Molecular Research Center for Children's Mental Development, United Graduate School of Child Development, Osaka University, Osaka, 565-0871, Japan; 174 Institute of Diagnostic Radiology and Neuroradiology, University Medicine Greifswald, 17489 Greifswald, Germany; 175 German Center for Neurodegenerative Diseases (DZNE), Tübingen, 72076, Germany; 176 Department of Psychology, VU University Amsterdam, 1081 BT Amsterdam, The Netherlands; 177 Department of Psychiatry, University of Iowa, Iowa City, Iowa 52242, USA; 178 Department of Epidemiology, Harvard T.H. Chan School of Public Health, Boston, Massachusetts 02115 USA; 179 HMNC Brain Health, Munich, 80539, Germany; 180 Interfaculty Institute for Genetics and Functional Genomics, University Medicine Greifswald, 17489 Greifswald, Germany; 181 Translational Genomics Research Institute, Neurogenomics Division, 445N Fifth Street, Phoenix, Arizona 85004, USA; 182 Department of Psychiatry, Fujita Health University School of Medicine, Toyoake 470-1192, Japan; 183 Department of Radiology, Mayo Clinic, Rochester, Minnesota 55905, USA; 184 NICHD Brain and Tissue Bank for Developmental Disorders, University of Maryland Medical School, Baltimore, Maryland 21201, USA; 185 School of Psychology, University of Sussex, Brighton BN1 9QH, UK; 186 Institute of Cognitive Neuroscience, University College London, London WC1N 3AR, UK; 187 Department of Neuroinformatics, Araya Brain Imaging, Tokyo, 102-0093, Japan; 188 Medical University of Lodz, 90-419 Lodz, Poland; 189 Department of Neurology, Mayo Clinic, Rochester, Minnesota, 55905, USA; 190 Maryland Psychiatric Research Center, Department of Psychiatry, University of Maryland School of Medicine, Baltimore, Maryland, 21228, USA; 191 Neuroscience Research Australia, Sydney, New South Wales 2031, Australia; 192 School of Medical Sciences, UNSW, Sydney, New South Wales 2052, Australia; 193 INSERM UMR 1000 “Neuroimaging and Psychiatry”, Service Hospitalier Frédéric Joliot; University Paris-Sud, Université Paris-Saclay, University Paris Descartes, Maison de Solenn, Paris, 91400, France; 194 Columbia University Medical Center, New York, New York 10032, USA; 195 Laboratory of Genetics, National Institute on Aging, National Institutes of Health, Baltimore, Maryland 21224, USA; 196 Departments of Neurology and Psychiatry, University of Pittsburgh, 3501 Forbes Ave., Suite 830, Pittsburgh Pennsylvania 15213, USA; 197 Department of Psychiatry, University of Oxford, Oxford OX3 7JX, UK; 198 Department of Radiology, Johns Hopkins University School of Medicine, Baltimore, Maryland 21205, USA; 199 Centre for Advanced Imaging, University of Queensland, Brisbane, Queensland 4072, Australia; 200 Section of Gerontology and Geriatrics, Department of Medicine, University of Perugia, 06132 Perugia, Italy; 201 Munich Cluster for Systems Neurology (SyNergy), 81377 Munich, Germany; 202 University of Liverpool, Institute of Translational Medicine, Liverpool L69 3BX, UK; 203 Institute of Clinical Chemistry and Laboratory Medicine, University Medicine Greifswald, 17489 Greifswald, Germany; 204 German Center for Cardiovascular Research (DZHK e.V.), partner site Greifswald, Greifswald, 17475, Germany; 205 Department of Statistics & WMG, University of Warwick, Coventry CV4 7AL, UK; 206 Department of Medical Informatics Erasmus MC, 3015 CE Rotterdam, The Netherlands; 207 Faculty of Applied Sciences, Delft University of Technology, Delft, 2628 CD, The Netherlands; 208 Department of Genomics, Life & Brain Center, University of Bonn, 53127 Bonn, Germany; 209 Rotman Research Institute, University of Toronto, Toronto, Ontario, Canada M6A 2E1; 210 Departments of Psychology and Psychiatry, University of Toronto, Toronto, Ontario, Canada M5T 1R8; 211 Child Mind Institute, New York, New York, 10022, USA; 212 Departments of Physiology and Nutritional Sciences, University of Toronto, Toronto, Ontario, Canada M5S 3E2; 213 Department of Radiology, University of Calgary, Calgary, Alberta, Canada T2N 4N1; 214 Department of Clinical Neuroscience, University of Calgary, Calgary, Alberta, Canada T2N 4N1; 215 Departments of Epidemiology, Medicine and Health Services, University of Washington, Seattle, WA, USA Group Health Research Institute, Group Health, 1730 Minor Avenue/Suite 1360, Seattle, Washington 98101, USA; 216 Department of Developmental Disability Neuropsychiatry, School of Psychiatry, University of New South Wales, Sydney, New South Wales 2052, Australia; 217 Institute for Translational Genomics and Population Sciences, Los Angeles Biomedical Research Institute and Pediatrics at Harbor-UCLA Medical Center, Torrance, California 90502, USA; 218 Evelyn F. McKnight Brain Institute, University of Miami, Miller School of Medicine, Miami, Florida, 33136, USA; 219 Neuropsychiatric Institute, Prince of Wales Hospital, Randwick, New South Wales 2031, Australia; 220 Institute of Molecular Biology and Biochemistry, Medical University Graz, Harrachgasse 21/III, 8010 Graz, Austria; 221 Department of Neuroimaging, Institute of Psychiatry, King's College London, London SE5 8AF, UK; 222 Biomedical Research Centre for Mental Health, King's College London, London SE5 8AF, UK; 223 Biomedical Research Unit for Dementia, King's College London, London SE5 8AF, UK; 224 MRC Edinburgh Brain Bank, University of Edinburgh, Academic Department of Neuropathology, Centre for Clinical Brain Sciences, Edinburgh, EH16 4SB UK; 225 Institute of Clinical Medicine, Neurology, University of Eastern Finland, FI-70211 Kuopio, Finland; 226 Neurocentre Neurology, Kuopio University Hospital, FI-70211 Kuopio, Finland; 227 Institute of Cardiovascular and Medical Sciences, Faculty of Medicine, University of Glasgow, Glasgow, G4 0SF, UK; 228 Laboratory of Neuro Imaging, Institute for Neuroimaging and Informatics, Keck School of Medicine of the University of Southern California, Los Angeles, California 90033, USA; 229 Department of Pathology, Johns Hopkins University, Baltimore, Maryland 21205, USA; 230 3rd Department of Neurology, "G. Papanicolaou", Hospital, Aristotle University of Thessaloniki, Thessaloniki, 57010, Greece; 231 Univ. Bordeaux, Inserm, Bordeaux Population Health Research Center, UMR1219, Bordeaux, F-33000, France; 232 Department of Internal Medicine, Erasmus MC, 3015 CE Rotterdam, The Netherlands; 233 Genentech Inc., South San Francisco, California 94080, USA; 234 Department of Psychiatry and Leiden Institute for Brain and Cognition, Leiden University Medical Center, 2333 ZA Leiden, The Netherlands; 235 University of Groningen, University Medical Center Groningen, Department of Neuroscience, 9713 AW Groningen, the Netherlands; 236 Department of Internal Medicine and Geriatric Medicine, INSERM U1027, University of Toulouse, Toulouse, 31024, France; 237 Department of Psychiatry, Carver College of Medicine, University of Iowa, Iowa City, Iowa 52242, USA; 238 Departments of Psychiatry, Neurology, Neuroscience and the Institute of Genetic Medicine, Johns Hopkins University School of Medicine, Baltimore, Maryland 21205, USA; 239 Center for Imaging of Neurodegenerative Disease, San Francisco VA Medical Center, University of California, San Francisco, California 94121, USA; 240 Department of Neurobiology, Care Sciences and Society, Karolinska Institutet, SE-141 57 Huddinge, Sweden; 241 Department of Child and Adolescent Psychiatry/Psychology, Erasmus MC-Sophia Children's Hospital, 3015 CE Rotterdam, The Netherlands; 242 Laboratory of Epidemiology & Population Sciences, National Institute on Aging, National Institutes of Health, Bethesda, Maryland 20892, USA; 243 Departments of Neurology and Epidemiology, University of Washington, 325 Ninth Avenue, Seattle, Washington 98104-2420, USA; 244 Intramural Research Program, NIA, NIH, 7201 Wisconsin Ave, Suite 3C-309, Bethesda, Maryland 20892, USA; 245 Department of Neurology, Erasmus MC, Rotterdam 3015 CE, The Netherlands

## Abstract

The hippocampal formation is a brain structure integrally involved in episodic memory, spatial navigation, cognition and stress responsiveness. Structural abnormalities in hippocampal volume and shape are found in several common neuropsychiatric disorders. To identify the genetic underpinnings of hippocampal structure here we perform a genome-wide association study (GWAS) of 33,536 individuals and discover six independent loci significantly associated with hippocampal volume, four of them novel. Of the novel loci, three lie within genes (*ASTN2*, *DPP4* and *MAST4*) and one is found 200 kb upstream of *SHH*. A hippocampal subfield analysis shows that a locus within the *MSRB3* gene shows evidence of a localized effect along the dentate gyrus, subiculum, CA1 and fissure. Further, we show that genetic variants associated with decreased hippocampal volume are also associated with increased risk for Alzheimer's disease (*r*_g_=−0.155). Our findings suggest novel biological pathways through which human genetic variation influences hippocampal volume and risk for neuropsychiatric illness.

## Abstract (web summary)

The hippocampus in mammalian brain varies in size across individuals. Here, Hibar and colleagues perform a genome-wide association meta-analysis to find six genetic loci with significant association to hippocampus volume.

Brain structural abnormalities in the hippocampal formation are found in many complex neurological and psychiatric disorders including temporal lobe epilepsy^1^, vascular dementia^2^, Alzheimer's disease^3^, major depression^4^, bipolar disorder^5^, schizophrenia^6^ and post-traumatic stress disorder^7^, among others. The diverse functions of the hippocampus, including episodic memory^8^, spatial navigation^9^, cognition^10^ and stress responsiveness^11^ are commonly impaired in a broad range of diseases and disorders of the brain that are associated with insults to the hippocampal structure. Further, the cytoarchitectural subdivisions (or ‘subfields') of the hippocampus are associated with distinct functions. For example, the dentate gyrus (DG) and sectors 3 and 4 of the cornu ammonis (CA) are involved in declarative memory acquisition^12^, the subiculum and CA1 play a role in disambiguation during working memory processes^13^, and the CA2 is implicated in animal models of episodic time encoding^14^ and social memory^15^. The anterior hippocampus, which includes the fimbria, CA subregions and hippocampal -amygdaloid transition area (HATA), may be involved in the mediation of cognitive processes including imagination, recall and visual perception^16^ and anxiety-related behaviours^17^.

Environmental factors, such as stress, affect the hippocampus^18^, but genetic differences across individuals account for most of the population variation in its size; the heritability of hippocampal volume is high at around 70% (refs 19, 20, 21). High heritability and a crucial role in healthy and diseased brain function make the hippocampus an ideal target for genetic analysis. We formed a large global partnership to empower the quest for mechanistic insights into neuropsychiatric disorders associated with hippocampal abnormalities and to chart, in depth, the genetic underpinnings of the hippocampal structure.

Here we perform a GWAS meta-analysis of mean bilateral hippocampal volume in 33,536 individuals scanned at 65 sites around the world as a joint effort between the Enhancing Neuroimaging Genetics through Meta-analysis (ENIGMA) and the Cohorts for Heart and Aging Research in Genomic Epidemiology (CHARGE) consortia. Our primary goal is to find common genetic determinants of hippocampal volume with previously unobtainable power. We make considerable efforts to coordinate data analysis across all sites from both consortia to maximize the comparability of both genetic and imaging data. Standardized protocols for image analysis and genetic imputation are freely available online (see URLs). In the most powerful imaging study of the hippocampus to date, we shed light on the common genetic determinants of hippocampal structure and allow for a deepened understanding of the biological workings of the brain's memory centre. We confirm previously identified loci influencing hippocampal volume, identify four novel loci and determine genome-wide overlap with Alzheimer's disease.

## Results

### Novel genome-wide markers associated with hippocampal volume

**Figure 1:**
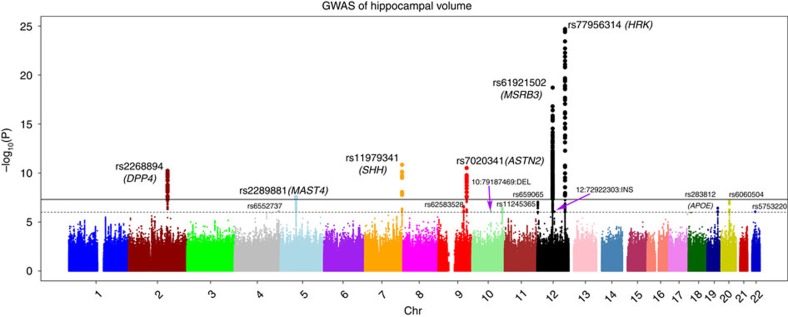
**Common genetic variants associated with hippocampal volume (*****N*****=26,814 of European ancestry).** A Manhattan plot displays the association *P* value for each single-nucleotide polymorphism (SNP) in the genome (displayed as –log_10_ of the *P*-value). Genome-wide significance is shown for the *P*=5 × 10^−8^ threshold (solid line) and also for the suggestive significance threshold of *P*=1 × 10^−6^ (dotted line). The most significant SNP within an associated locus is labeled. For the significant loci and age-dependent loci (Chromosome 19) we labeled the nearest gene, which is not necessarily the gene of action.

**Figure 2:**
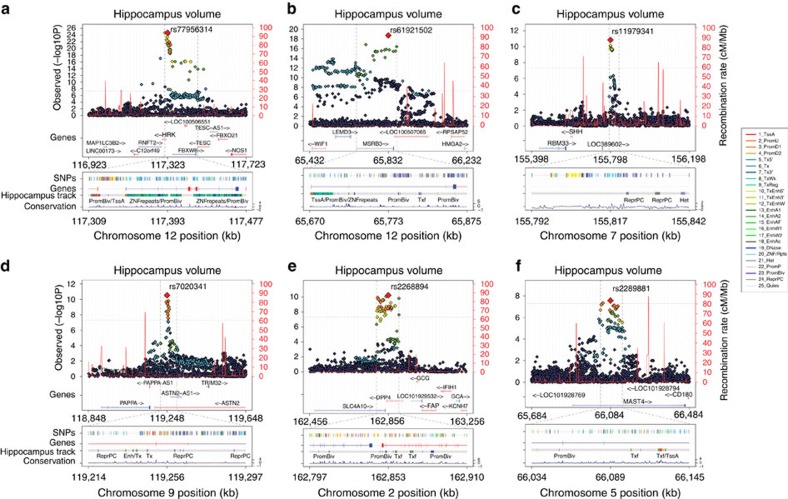
Functional annotation within genome-wide significant loci. For each panel (**a**–**f**), zoomed-in Manhattan plots (±400 kb from top SNP) are shown with gene models below (GENCODE version 19). Plots below are zoomed to highlight the genomic region that likely harbors the causal variant(s) (*r*^2^>0.8 from the top SNP). Genomic annotations from the Roadmap Epigenomics Consortium^53^ are displayed to indicate potential functionality (see Methods for detailed track information). Each plot was made using the LocusTrack software^55^ (see URLs).

Our combined meta-analysis (*n*=26,814 individuals of European ancestry) revealed six independent, genome-wide significant loci associated with hippocampal volume (Fig. 1; Table 1). Four are novel: with index SNPs rs11979341 (7q36.3; *P*=1.42 × 10^−11^), rs7020341 (9q33.1; *P*=3.04 × 10^−11^), rs2268894 (2q24.2; *P*=5.89 × 10^−11^), and rs2289881 (5q12.3; *P*=2.73 × 10^−8^). The other two loci have been previously characterized in detail: with index SNPs rs77956314 (12q24.22, *P*=2.06 × 10^−25^), in linkage disequilibrium (LD) (*r*^2^=0.901 in European samples from the 1000 Genomes Project, Phase 1v3) with our previously identified variant at this locus (rs7294919) and rs61921502 (12q14.3, *P*=1.94 × 10^−19^), in LD (*r*^2^=0.459) with previous top locus rs17178006 (refs 22, 23, 24; Fig. 2a–f). In addition to these SNPs, we identified nine independent loci with a statistically suggestive influence on hippocampal volume (*P*<1 × 10^−6^; Supplementary Data 4). All pathway results and gene-based *P* values are summarized in Supplementary Data 6 and 7.

### Variance explained in hippocampal volume by common variants

**Figure 3:**
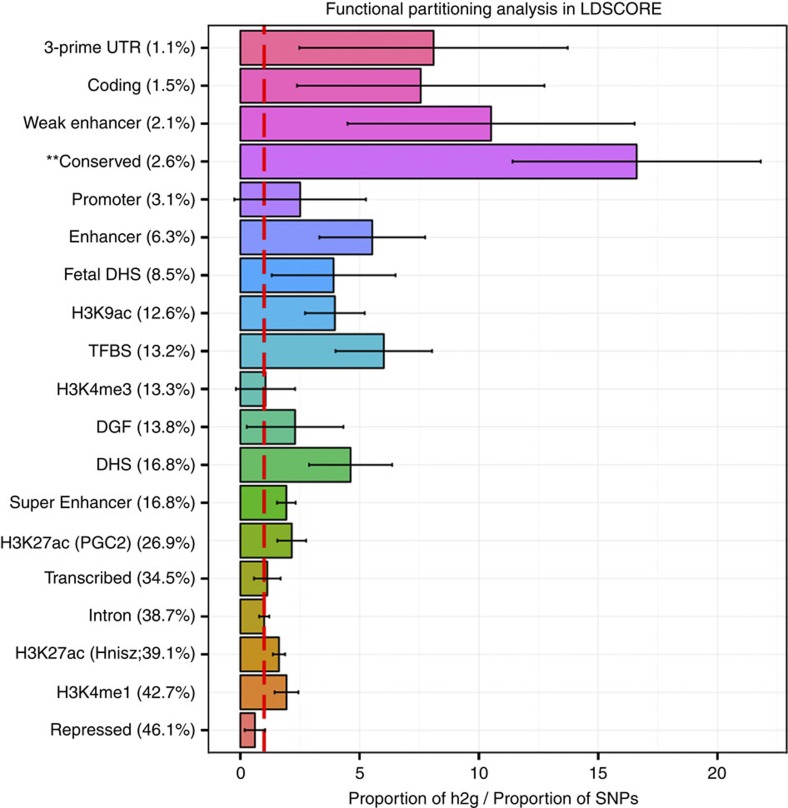
Analysis of variance explained, functional annotation, and pathway analysis. LDSCORE regression analysis for different functional annotation^26^ categories (described further in Finucane *et al*.^26^). Plotted values are the proportion of *h*^2^_g_ explained divided by the proportion of SNPs in a given functional category. Values are significantly over- or under-represented if they differ significantly from 1. Values are plotted with a standard error calculated with a jackknife in LDSCORE. Evolutionarily conserved regions across mammals significantly contributed to the heritability of hippocampal volume (indicated by **).

Common variants genotyped from across the whole-genome explained as much as 18.76% (s.e. 1.56%) of the observed variance in human hippocampal volume, based on LDSCORE regression^25^ (Supplementary Fig. 3). Common genetic variants account for around a quarter of the overall heritability, estimated in twin studies to be around 70% (refs 19, 20, 21). Further partitioning the genome into functional categories using LDSCORE^26^ revealed significant over-representation of regions evolutionarily conserved in mammals (*P*=0.0026): 2.6% of the variants accounted for 43.3% of the 18.76% variance explained (Fig. 3).

### Effects of top variants on hippocampal subfield volume

To test for differential effects on individual subfields of the hippocampal formation, we examined the six significant variants influencing whole hippocampal volume in a large cohort (*n*=5,368). We found that the top SNP from our primary analysis, rs77956314, has a broad, nonspecific effect on hippocampal subfield volumes with the greatest effect in the right hippocampal tail (*P*=1.27 × 10^−8^). rs61921502 showed strong lateral effects across right hippocampal subfields with the largest effect in the right hippocampal fissure (*P*=6.45 × 10^−9^). rs7020341 showed greatest effects bilaterally in the subiculum (left: *P*=1.59 × 10^−8^; right: *P*=1.42 × 10^−8^). rs2268894 show left-lateralized effects across hippocampal subfields with the strongest effect in the left hippocampal tail (*P*=1.76 × 10^−5^). The remaining two variants (rs11979341 and rs2289881) did not show significant evidence of association across any of the hippocampal subfields. The full set of results from the hippocampal subfield analysis is tabulated in Supplementary Data 8.

### Genetic overlap with hippocampal volume

We used LDSCORE^27^ regression to quantify the degree of common genetic overlap between variants influencing the hippocampus and those influencing Alzheimer's disease. We found significant evidence of a moderate, negative relationship whereby variants associated with a decrease in hippocampal volume are associated with an increased risk for Alzheimer's disease (*r*_g_=−0.155 (s.e. 0.0529), *P*=0.0034; see Methods).

## Discussion

We identified six genome-wide significant, independent loci associated with hippocampal volume in 26,814 subjects of European ancestry. Of the six loci, four were novel: rs11979341 (7q36.3; *P*=1.42 × 10^−11^), rs7020341 (9q33.1; *P*=3.04 × 10^−11^), rs2268894 (2q24.2; *P*=5.89 × 10^−11^) and rs2289881 (5q12.3; *P*=2.73 × 10^−8^). We previously discovered two of the novel loci, rs7020341 and rs2268894 (ref. 24), but in this higher-powered analysis they now surpassed the genome-wide significance. In addition to the four novel loci, we replicated two loci associated with hippocampal volume: rs7492919 and rs17178006 (refs 23, 24). Hibar *et al*.^22^ previously reported additional support for the rs17178006 association with hippocampal volume.

Each novel locus identified has unique functions and has previously been linked to diseases of the brain. Variant rs7020341 lies within an intron of the *astrotactin 2* (*ASTN2*) gene (Fig. 2d) which encodes for a protein involved in glial-mediated neuronal migration in the developing brain^28^. Rare deletions overlapping this locus near the 3′ end of *ASTN2* have been observed in patients with autism spectrum disorder and attention-deficit/hyperactivity disorder^29^. Common variants near this site are associated with autism spectrum disorders^29^ and migraine^30^. Variant rs2268894 is located in an intron of *DPP4* (Fig. 2e) that encodes dipeptidyl peptidase IV; an enzyme regulating response to the ingestion of food^31^, and an established target of a treatment for type 2 diabetes mellitus (vildagliptin)^32^. In addition, rs2268894 is in strong LD (*r*^2^=0.83) with a genome-wide significant locus associated with a decreased risk for schizophrenia (rs2909457)^33^; however, the allele that increases risk for schizophrenia also increases hippocampal volume even though patients with schizophrenia show decreased hippocampal volume relative to controls^6^. Variant rs11979341 lies in an intergenic region (Fig. 2c) around 200 kb upstream of the *sonic hedgehog* (*SHH*) gene, crucial for neural tube formation^34^. Adult brain expression data provide some evidence that rs11979341-C increases the expression of *SHH* in adult human hippocampus^35^ (*P*=0.0089). Finally, variant rs2289881 lies within an intron of the microtubule-associated serine/threonine kinase family member 4 (*MAST4*) gene (Fig. 2f). The protein product of *MAST4* modulates the microtubule scaffolding; the gene has been linked to susceptibility for atherosclerosis in HIV-infected men^36^, and atypical frontotemporal dementia^37^.

Effect sizes from the full sample were almost identical to those obtained from a subset meta-analysis (Pearson's *r*^2^>0.99; *n*=22,761) that removed all patients diagnosed with a neuropsychiatric disorder. Observed effects are therefore not likely to be driven by inclusion of patients with brain disorders. All significant loci are tabulated in Table 1. We found little evidence that these effects could be generalized to populations of African, Japanese, and Mexican-American ancestry, which could be due to the limited power from smaller non-European sample sizes available (*n*=6,722; Supplementary Data 5).

We estimated that 18.76% (s.e. 1.56%) of the variance in hippocampal volume could be explained by genotyped common genetic variation. This effect was only tested within populations of European ancestry and does not necessarily reflect the level of explained variance in other populations worldwide. This is a substantial fraction of the overall genetic component of variance determined by twin heritability studies, and the heritability of hippocampal volume is relatively high at around 70% (refs 19, 20, 21). With the same LDSCORE method, we estimated the amount of variance explained by common gene variants belonging to known functional cell categories^26^. We discovered enrichment of genomic regions conserved across mammals, which may have a strong evolutionary role in the hippocampal formation, a structure much more extensively developed in mammals than in other vertebrates^38^. Given that hippocampal atrophy is a hallmark of Alzheimer's disease pathology^39^, we were motivated to examine common genetic overlap between hippocampal volume and Alzheimer's disease risk. We found a significant negative relationship (*r*_g_=−0.155 (s.e. 0.0529), *P*=0.0034), through which loci associated with decreased hippocampal volume also increase risk for AD. This confirms a shared etiological component between AD and hippocampal volume whereby genetic variants influencing hippocampal volume also modify the risk for developing AD.

As the hippocampal formation is a complex structure comprised of diverse functional units, we sought to examine the genetic variants identified in our analysis for focal effects on hippocampal subfield volumes. When assessing 13 subfields of the hippocampus (26 total, left and right) we found that two of the top variants from our analysis (rs77956314 and rs7020341) had largely non-specific effects: most of the subfield volumes showed significant evidence of association (Supplementary Data 8). The variant rs61921502 showed a lateralized effect across the body of the right hippocampal formation, which includes the DG, subiculum, CA1 and fissure. Volume losses are frequently observed across the hippocampal body in AD^40^, major depression^41^, bipolar disorder^42^ and temporal lobe epilepsy^43^. Prior pathway analyses have implicated the rs61921502 with *MSR3B*, a gene related to oxidative stress^24^. Genetic variation at *MSR3B* may influence neurogenesis specifically within the dentate regions of the hippocampal body, where cell proliferation is known to continue into adulthood in healthy humans^44^. However, further functional validation is required to test this hypothesis. Finally, the variant rs2268894 was associated with volume differences in the left hippocampal tail, a subfield that has previously shown shape abnormalities^45^ and volume differences^46^ in schizophrenia.

Here we identified four novel loci associated with hippocampal volume and examined each variant for localized effects in hippocampal subfields. When partitioning the full genome-wide association results into functionally annotated categories, we discovered that SNPs in evolutionarily conserved regions were significantly over-represented in their contribution to hippocampal volume. Further, we found significant evidence of shared genetic overlap between hippocampal volume and Alzheimer's disease. This large international effort shows that by mapping out the genetic influences on brain structure, we may begin to derive mechanistic hypotheses for brain regions causally implicated in the risk for neuropsychiatric disorders.

## Methods

### Subjects and sites

High-resolution MRI brain scans and genome-wide genotyping data were available for 33,536 individuals from 65 sites in two large consortia: the ENIGMA Consortium and the CHARGE Consortium. Full details and demographics for each participating cohort are given in Supplementary Data 1. All participants (or their legal representatives) provided written informed consent. The institutional review board of the University of Southern California and the local ethics board of Erasmus MC University Medical Center approved this study.

### Imaging analysis and quality control

Hippocampal volumes were estimated using the automated and previously validated segmentation algorithms, FSL FIRST^47^ from the FMRIB Software Library (FSL) and FreeSurfer^48^. Hippocampal segmentations were visually examined at each site, and poorly segmented scans were excluded. Sites also generated histogram plots to identify any volume outliers. Individuals with a volume more than three standard deviations away from the mean were visually inspected to verify proper segmentation. Statistical outliers were included in analysis if they were properly segmented; otherwise, they were removed. Average bilateral hippocampal volume was highly correlated across automated procedures used to measure it (Pearson's *r*=0.74)^22^. A measure of head size—intracranial volume (ICV)—was used as a covariate in these analyses to adjust for volumetric differences due to differences in head size alone. Most sites measured ICV for each participant using the inverse of the determinant of the transformation matrix required to register the subject's MRI scan to a common template and then multiplied by the template volume (1,948,105 mm^3^). Full details of image acquisition and processing performed at each site are given in Supplementary Data 2.

### Genetic imputation and quality control

Genetic data were obtained at each site using commercially available genotyping platforms. Before imputation, genetic homogeneity was assessed in each sample using multi-dimensional scaling (MDS). Ancestry outliers were excluded by visual inspection of the first two components. The primary analysis and all data presented in this main text were derived from subjects with European ancestry. Replication attempts in subjects of additional ancestries are presented in Supplementary Data 5. Data were further cleaned and filtered to remove single-nucleotide polymorphisms (SNPs) with low minor allele frequency (MAF<0.01), deviations from Hardy–Weinberg Equilibrium (HWE; *P*<1 × 10^−6^), and poor genotyping call rate (<95%). Cleaned and filtered datasets were imputed to the 1000 Genomes Project reference panel (phase 1, version 3) using freely available and validated imputation software (MaCH/minimac, IMPUTE2, BEAGLE, GenABLE). After imputation, genetic data were further quality checked to remove poorly imputed SNPs (estimated *R*^2^<0.5) or low MAF (<0.5%). Details on filtering criteria, quality control, and imputation at each site may be found in Supplementary Data 3.

### Genome-wide association analysis and statistical models

GWAS were performed at each site, as follows. Mean bilateral hippocampal volume ((left+right)/2) was the trait of interest, and the additive dosage value of a SNP was the predictor of interest, while controlling for 4 MDS components, age, age^2^, sex, intracranial volume and diagnosis (when applicable). For studies with data collected from multiple centres or scanners, additional covariates were also included in the model to adjust for any scanning site effects. Sites with family data (NTR-Adults, BrainSCALE, QTIM, SYS, GOBS, ASPSFam, ERF, GeneSTAR, NeuroIMAGE, OATS, RSIx) used mixed-effects models to account for familial relationships, in addition to covariates stated previously. The primary analyses for this paper focused on the full set of individuals, including datasets with patients, to maximize power. We re-analysed the data excluding patients to verify that detected effects were not due to disease alone. The regression coefficients for SNPs with *P*<1 × 10^−5^ in the model including all patients were almost perfectly correlated with the regression coefficients from the model including only healthy individuals (Pearson's *r*=0.996). Full details for the software used at each site are given in Supplementary Data 3.

The GWAS of mean hippocampal volume was performed at each site, and the resulting summary statistics uploaded to a centralized site for meta-analysis. Before meta-analysis, GWAS results from each site were checked for genomic inflation and errors using Quantile–Quantile (QQ) plots (Supplementary Figs 1 and 2). GWAS results from each site were combined using a fixed-effects sample size-weighted meta-analysis framework as implemented in METAL^49^. Data were meta-analysed first in the ENIGMA and CHARGE Consortia separately and then combined into a final meta-analysed result file. After the final meta-analysis, SNPs were excluded if the SNP was available for fewer than 5,000 individuals.

### Variance explained and genetic overlap in hippocampal volume

The common genetic overlap, total variance explained by the GWAS, and the partitioned heritability analyses were estimated using LDSCORE^25^,^26^. Following from the polygenic model, an association test statistic at a given locus includes signal from all linked loci. Given a heritable polygenic trait, a SNP in high LD with, or tagging, a large number of SNPs is on average likely to show stronger association than a SNP that is not. The magnitude of information conveyed by each variant (a function of the number of SNPs tagged taking into account the strength of the tagging) is summarized as an LD score. By regressing the LD scores on the test statistics, we estimated the proportion of variance in the trait explained by the variants included in the analysis. As an extension, two LD score models for two separate traits can be used to estimate the covariance (and correlation) structure to yield an estimate of the common genetic overlap (*r*_g_) between any two trait pairs. Here we estimated the common genetic overlap between hippocampal volume and Alzheimer's disease^50^. Standard errors were estimated using a block jackknife.

### Genomic partitioning into functional categories

As well as estimating the total variance explained, the genomic heritability (*h*^2^_g_) can be partitioned into specific subsets of variants. The functional annotation partitioning used the pre-prepared LDSCORE and annotation (.annot) files available online (see URLs) following the method of Finucane *et al*.^26^. These analyses use the following 24 functional classes not specifically unique to any cell type: coding, UTR, promoter, intron, histone marks H3K4me1, H3K4me3, H3K9ac5 and two versions of H3K27ac, open chromatin DNase I hypersensitivity Site (DHS) regions, combined chromHMM/Segway predictions, regions conserved in mammals, super-enhancers and active enhancers from the FANTOM5 panel of samples (Finucane *et al*., page 4)^26^. Annotated coordinates are determined by a combination of all cell types from ENCODE. As in Finucane *et al*.^26^, to avoid bias, we included the 500 bp windows surrounding the variants included in the functional classes. The chromosome-partitioned analyses were conducted using LDSCOREs calculated for each chromosome. Following the method of Bulik-Sullivan *et al*.^25^, these analyses focus on the variants within HapMap3 as these SNPs are typically well imputed across cohorts. Enrichment of a given partition is calculated as the proportion of *h*^2^_g_ explained by that partition divided by the proportion of variants in the GWAS that fall into that partition. All LDSCORE analyses used non-genomic controlled meta-analyses.

### Gene annotation and pathway analysis

Gene annotation, gene-based test statistics, and pathway analysis were performed using the KGG2.5 software package^51^ (Supplementary Data 6 and 7). LD was calculated based on RSID numbers using the 1000 Genomes Project European samples as a reference (see URLs). For annotation, SNPs were considered ‘within' a gene, if they fell within 5 kb of the 3′/5′ UTR based on human genome (hg19) coordinates. Gene-based tests were performed using the GATES test^51^ without weighting *P* values by predicted functional relevance. Pathway analysis was performed using the HYST test of association^52^. For all gene-based tests and pathway analyses, results were considered significant if they exceeded a Bonferroni correction threshold accounting for the number of pathways in the REACTOME database tested such that *P*_thresh_=0.05/(671 pathways)=7.45 × 10^−5^.

### Annotation of SNPs with epigenetic factors

In Fig. 2, all tracks were taken from the UCSC Genome Browser Human hg19 assembly. *SNPs (top 5%)* shows the top 5% associated SNPs within the locus and are coloured by their correlation to the top SNP. *Genes* shows the gene models from GENCODE version 19. *Hippocampus* gives the predicted chromatin states based on computational integration of ChIP-seq data for 18 chromatin marks in human hippocampal tissue derived from the Roadmap Epigenomics Consortium^53^. The 18 chromatin states from the *hippocampus* track are as follows: TssA (Active TSS), TssFlnk (Flanking Active TSS), TssFlnkU (Flanking TSS Upstream), TssFlnkD (Flanking TSS Downstream), Tx (Strong transcription), TxWk (Weak transcription), EnhG1 (Genic Enhancers 1), EnhG2 (Genic Enhancers 2), EnhA1 (Active Enhancers 1), EnhA2 (Active Enhancers 2), EnhWk (Weak Enhancers), ZNF/Rpts (ZNF genes & repeats), Het (Heterochromatin), TssBiv (Bivalent/Poised TSS), EnhBiv (Bivalent Enhancer), ReprPC (Repressed PolyComb), ReprPCWk (Weak Repressed PolyComb), Quies (Quiescent/Low). Additional information about the 18 state chromatin model is detailed elsewhere^53^. *Conservation* is the basewise conservation score over 100 vertebrates estimated by PhyloP from the UCSC Genome Browser Human hg19 assembly.

### Analysis of hippocampal subfields

We segmented the hippocampal formation into 13 subfield regions: CA1, CA3, CA4, fimbria, Granule Layer+Molecular Layer+Dentate Gyrus Boundary (GC_ML_DG), hippocampal-amygdaloid transition area (HATA), hippocampal tail, hippocampal fissure, molecular layer (HP), parasubiculum, presubiculum and subiculum using a freely available, validated algorithm distributed with the FreeSurfer image analysis package^54^. We measured the hippocampal subfield volumes within the Rotterdam (*n*=4,491) and HUNT (*n*=877) cohorts. Volumes from the 26 subfield regions (13 in each hemisphere) were the phenotypes of interest and individually assessed for significance with the top variants from our primary analysis while correcting for the following nuisance variables: 4 MDS components, age, age^2^, sex, intracranial volume. Association statistics from each of the tests in the Rotterdam and HUNT cohorts were meta-analysed using a fixed-effects inverse variance-weighted model yielding the final results. We declare an individual test significant if the *P* value is less than a Bonferroni-corrected *P* value threshold accounting for the total number of tests: *P*_thresh_=0.05/(26 subfields × 6 SNPs)=3.21 × 10^−4^.

### Data availability

The genome-wide summary statistics that support the findings of this study are available upon request from the corresponding authors MAI and PMT (see URLs). The data are not publicly available due to them containing information that could compromise research participant privacy/consent.

### URLs

https://github.com/bulik/ldsc

http://enigma.usc.edu/protocols/genetics-protocols/

http://gump.qimr.edu.au/general/gabrieC/LocusTrack/

http://enigma.ini.usc.edu/download-enigma-gwas-results/

http://www.internationalgenome.org/data

## Additional information

**How to cite this article:** Hibar, D. P. *et al*. Novel genetic loci associated with hippocampal volume. *Nat. Commun.* **8**, 13624 doi: 10.1038/ncomms13624 (2017).

**Publisher's note:** Springer Nature remains neutral with regard to jurisdictional claims in published maps and institutional affiliations.

## Supplementary Material

Supplementary Information Supplementary Figures 1-3, Supplementary Notes 1-3

Supplementary Data 1 Demographic description of cohorts included in the analysis

## Figures and Tables

**Table 1:** Genetic variants at six loci were significantly associated with hippocampal volume.

**RSID**	**Chr**	**Pos**	**Nearest gene**	**Allele1**	**Allele2**	**Freq**	***Z*****-score**	***N***	***P*** **value**
**rs77956314**	12	117,323,367	4 kb 5′ to *HRK*	T	C	0.9160	−10.418	26,814	2.06 × 10^−25^
**rs61921502**	12	65,832,468	Intron of *MSRB3*	T	G	0.8466	9.017	26,814	1.94 × 10^−19^
**rs11979341**	7	155,797,978	200 kb 5′ to *SHH*	C	G	0.6837	−6.755	24,484	1.42 × 10^−11^
**rs7020341**	9	119,247,974	Intron of *ASTN2*	C	G	0.3590	6.645	26,700	3.04 × 10^−11^
**rs2268894**	2	162,856,148	Intron of *DPP4*	T	C	0.5412	−6.546	26,814	5.89 × 10^−11^
**rs2289881**	5	66,084,260	Intron of *MAST4*	T	G	0.3544	−5.558	26,814	2.73 × 10^−8^

The allele frequency (Freq) and effect size (*Z*-score) are given with reference to Allele 1. Effect sizes are additive effects for each copy of Allele 1 given as a *Z*-score. Additional validation was attempted in non-European ancestry generalization samples (shown in Supplementary Data 5).
