# Supplementary material for: Novel genetic loci associated with hippocampal volume
Source: Nat Commun. 2017 Jan 18;8:13624. doi: 10.1038/ncomms13624 (PMC5253632; doi:10.1038/ncomms13624)
Supplement: Supplementary Information — Supplementary Figures 1-3, Supplementary Notes 1-3 [file ncomms13624-s1.pdf]

**Supplemental Figure 1 (a-b)**. (a) Quantile-Quantile plots of the GWAS of hippocampal volume results for each individual study from the ENIGMA Consortium (split into two panels a and b). Lambda inflation factors are provided for each plot. The red line represents the expected null distribution.

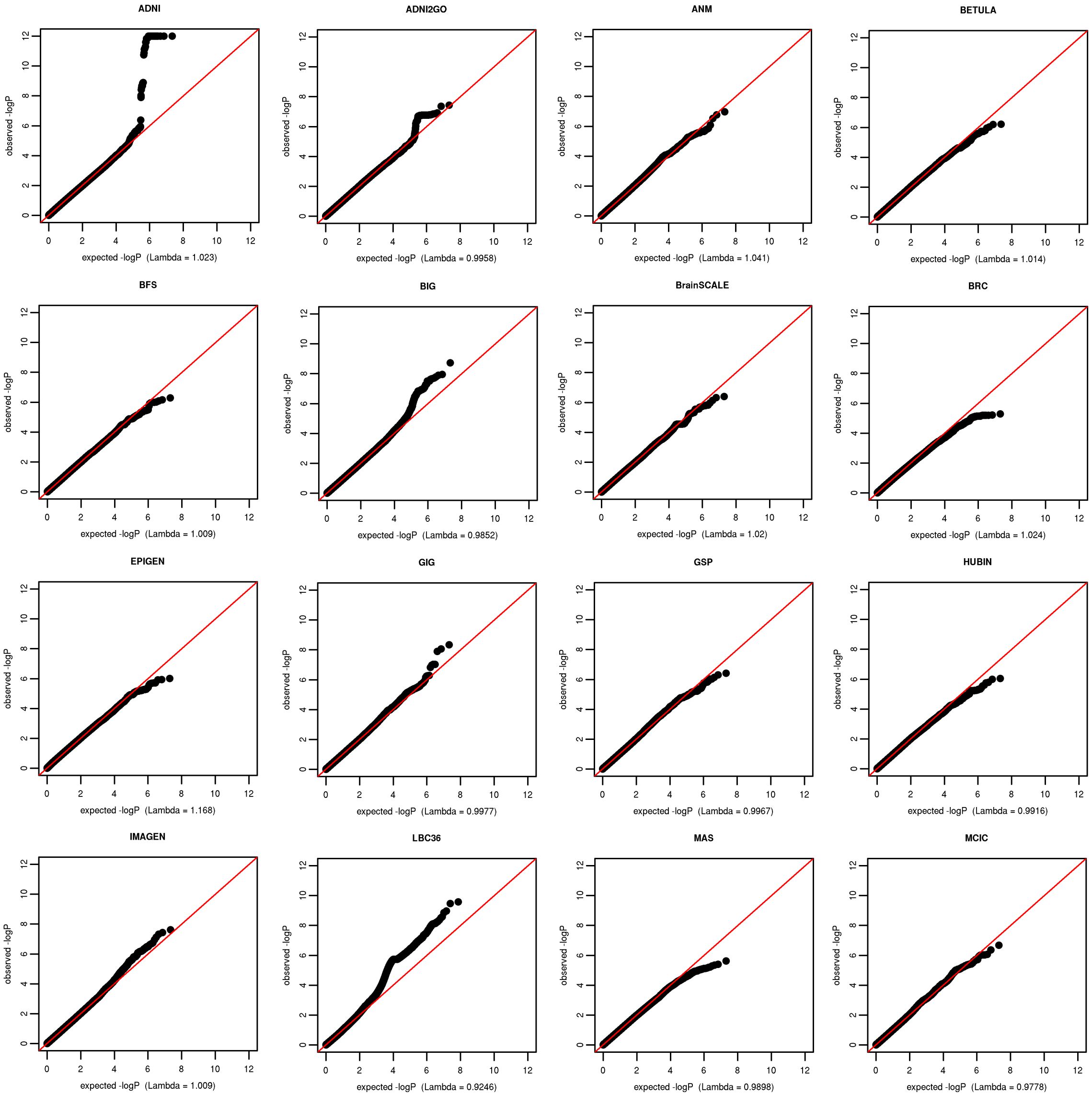

**Supplemental Figure 1 (a-b).** (b) Quantile-Quantile plots of the GWAS of hippocampal volume results for each individual study from the ENIGMA Consortium (split into two panels a and b). Lambda inflation factors are provided for each plot. The red line represents the expected null distribution.

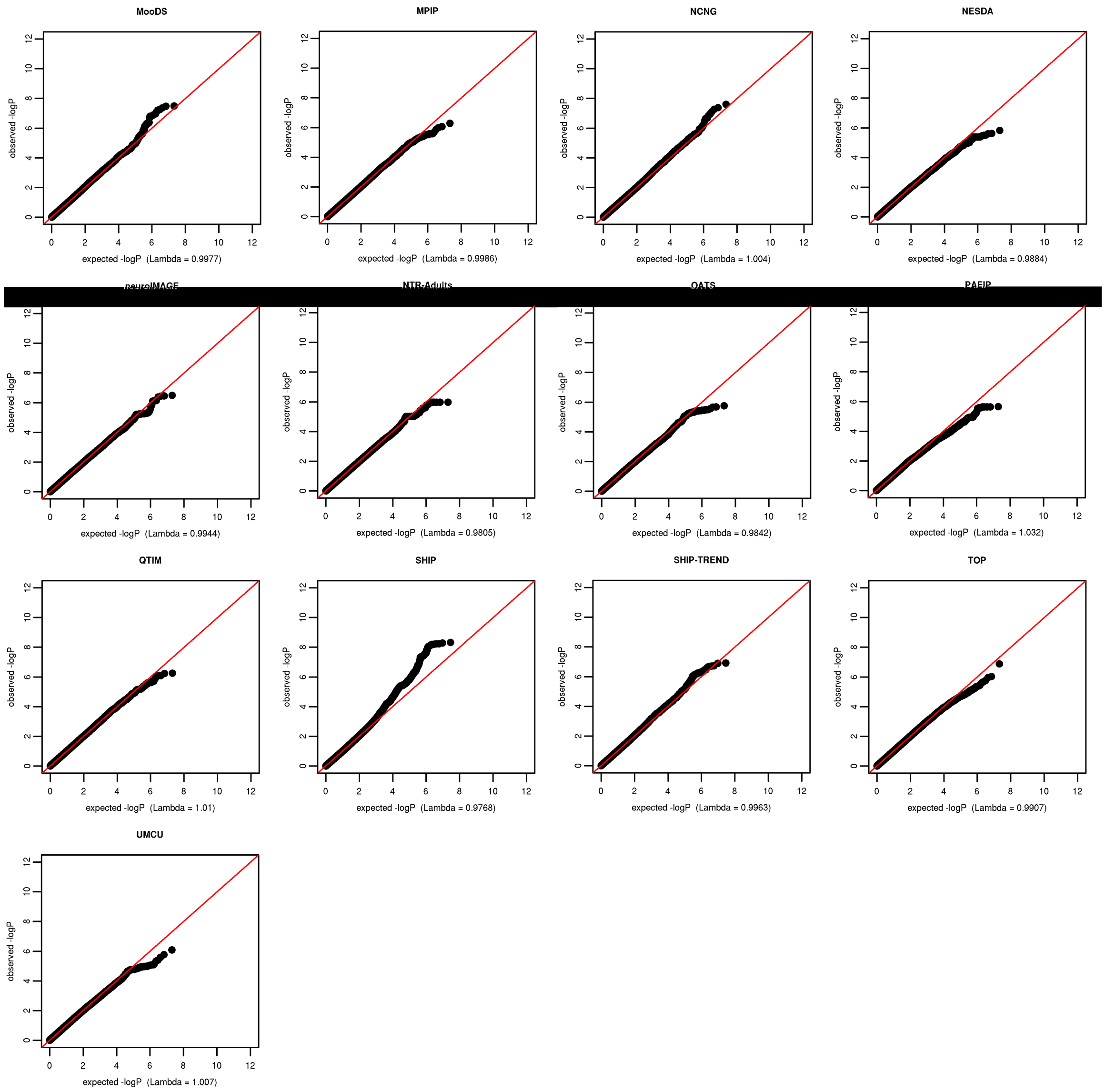

**Supplemental Figure 2.** (b) Quantile-Quantile plots of the GWAS of hippocampal volume results for each individual study from the CHARGE Consortium. Lambda inflation factors are provided for each plot. The red line represents the expected null distribution.

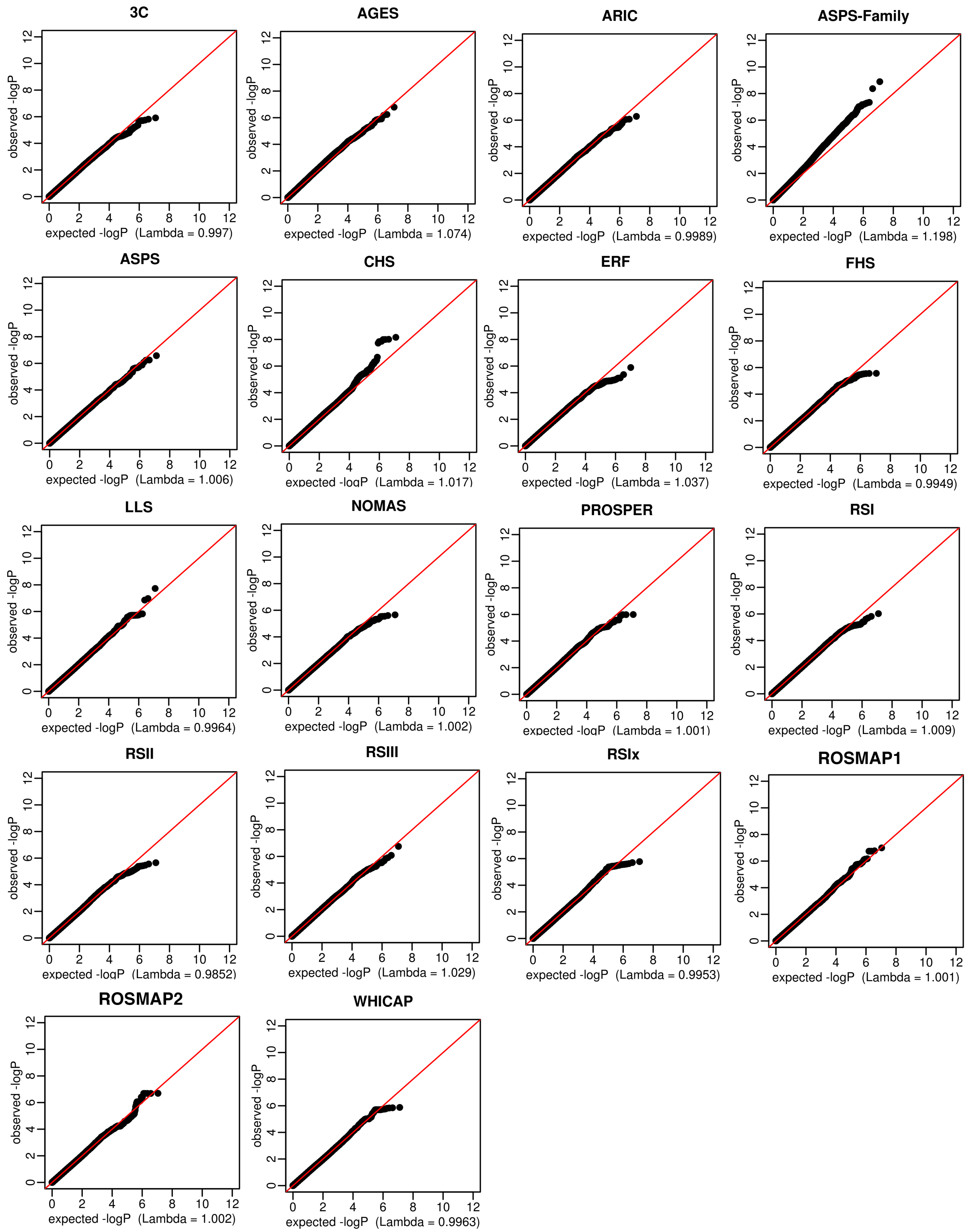

**Supplemental Figure 3.** LDSCORE regression analysis split by chromosome. Plotted values are the proportion of $h^2_g$ explained divided by the proportion of SNPs in a given chromosome. Values are significantly over- or under-represented if they differ significantly from 1. Values are plotted with a standard error calculated with a jackknife in LDSCORE. Chromosome 4 had a significant under-representation in its contribution to the overall heritability estimate (indicated by **).

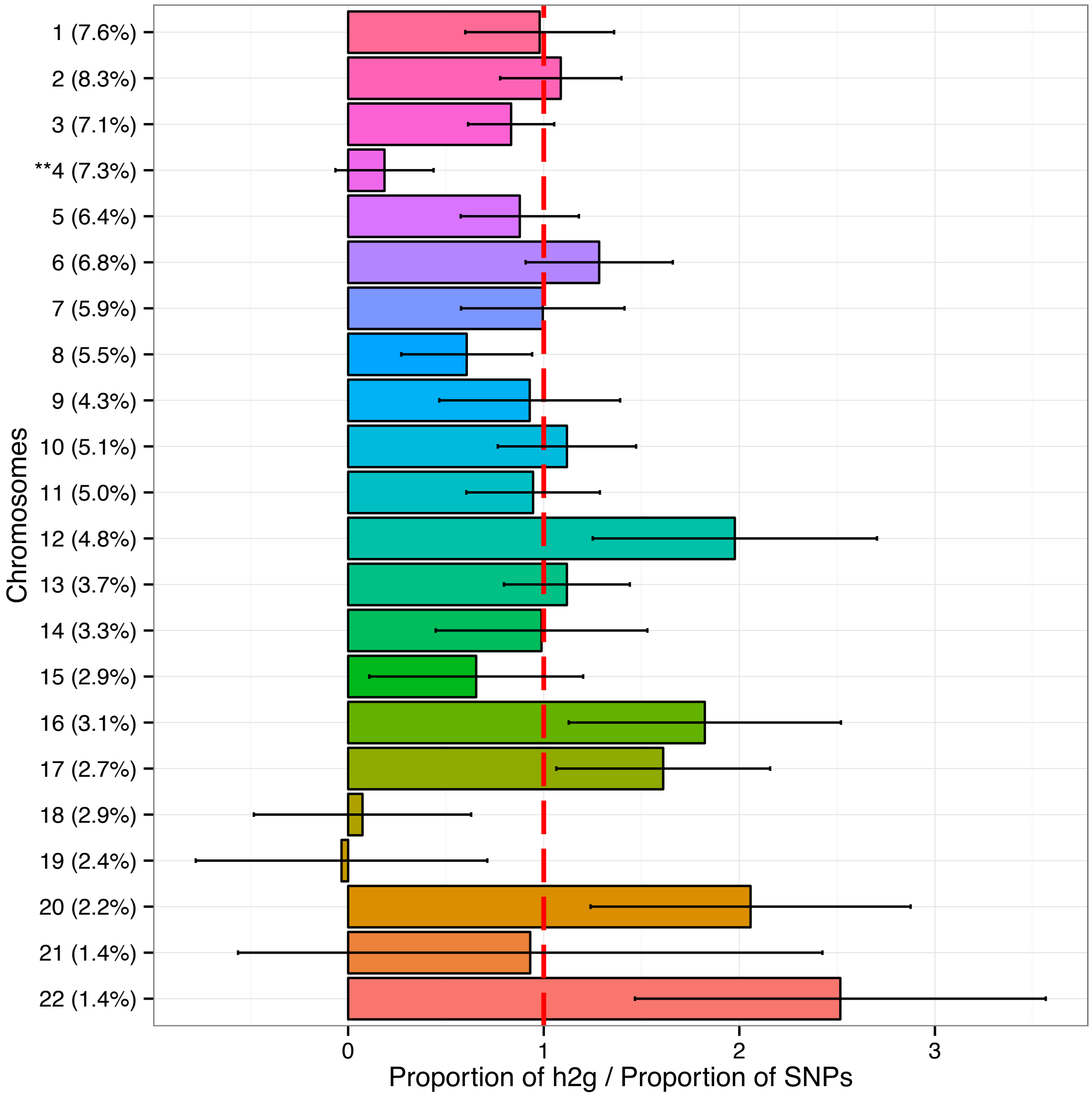

**Supplementary Note 1: Consortium Authors:**

**The following authors are included under the Alzheimer's Disease Neuroimaging Initiative (ADNI):**

Data used in preparing this article were obtained from the Alzheimer's Disease Neuroimaging Initiative (ADNI) database (adni.loni.usc.edu). As such, many investigators within the ADNI contributed to the design and implementation of ADNI and/or provided data but did not participate in analysis or writing of this report. A complete listing of ADNI investigators can be found at: http://adni.loni.usc.edu/wp-content/uploads/how_to_apply/ADNI_Acknowledgement_List.pdf

Michael Weiner (UC San Francisco), Paul Aisen (UC San Diego), Ronald Petersen (Mayo Clinic, Rochester), Clifford R. Jack, Jr. (Mayo Clinic, Rochester), William Jagust (UC Berkeley), John Q. Trojanowki (U Pennsylvania), Arthur W. Toga (USC), Laurel Beckett (UC Davis), Robert C. Green (Brigham and Women's Hospital / Harvard Medical School), Andrew J. Saykin (Indiana University), John Morris (Washington University St. Louis), Leslie M. Shaw (University of Pennsylvania); ADNI External Advisory Board (ESAB): Zaven Khachaturian (Prevent Alzheimer's Disease 2020), Greg Sorensen (Siemens), Maria Carrillo (Alzheimer's Association), Lew Kuller (University of Pittsburgh), Marc Raichle (Washington University St. Louis), Steven Paul (Cornell University), Peter Davies (Albert Einstein College of Medicine of Yeshiva University), Howard Fillit (AD Drug Discovery Foundation), Franz Hefti (Acumen Pharmaceuticals), Davie Holtzman (Washington University St. Louis), M. Marcel Mesulman (Northwestern University), William Potter (National Institute of Mental Health), Peter Snyder (Brown University); **ADNI 2 Private Partner Scientific Board (PPSB) Chair:** Adam Schwartz (Eli Lilly); **Data and Publication Committee (DPC)**: Robert C. Green (Brigham and Women's Hospital/Harvard Medical School (Chair)); **Resource Allocation Review Committee**: Tom Montine (University of Washington (Chair)); **Clinical Core Leaders:** Ronald Petersen (Mayo Clinic, Rochester), Paul Aisen (UC San Diego); **Clinical Informatics and Operations**: Ronald G. Thomas (UC San Diego), Michael Donohue (UC San Diego), Sarah Walter (UC San Diego), Devon Gessert (UC San Diego), Tamie Sather (UC San Diego), Gus Jiminez (UC San Diego); **Biostatistics Core Leaders and Key Personnel:** Laurel Beckett (UC Davis), Danielle Harvey (UC Davis), Michael Donohue (UC San Diego); **MRI Core Leaders and Key Personnel:** Clifford R. Jack, Jr. (Mayo Clinic, Rochester), Matthew Bernstein (Mayo Clinic, Rochester), Nick Fox (University of London), Paul Thompson (Keck School of Medicine of USC), Norbert Schuff (UCSF), Charles DeCarli (UC Davis), Bret Borowski (Mayo Clinic), Jeff Gunter (Mayo Clinic), Matt Senjem (Mayo Clinic), Prashanthi Vemuri (Mayo Clinic), David Jones (Mayo Clinic), Kejal Kantarci (Mayo Clinic), Chad Ward (Mayo Clinic); **PET Core Leaders and Key Personnel:** William Jagust (UC Berkeley)**,** Robert A. Koeppe (University of Michigan), Norm Foster (University of Utah)**,** Eric M. Reiman (Banner Alzheimer's Institute), Kewei Chen (Banner Alzheimer's Institute), Chet Mathis (University of Pittsburgh), Susan Landau (UC Berkeley); **Neuropathology Core Leaders:** John Morris (Washington University St. Louis), Nigel J. Cairns (Washington University St. Louis), Erin Householder (Washington University St. Louis), Lisa Taylor-Reinwald (Washington University St. Louis); **Biomarkers Core Leaders and Key Personnel:** J.Q. Trojanowki (UPenn School of Medicine)**,** Les Shaw (UPenn School of Medicine)**,** Virginia M.Y. Lee (UPenn School of Medicine)**,** Magdalena Korecka (UPenn School of Medicine), Michal Figurski (UPenn School of Medicine); **Informatics Core Leaders and Key Personnel:** Arthur W. Toga (USC), Karen Crawford (USC)**,** Scott Neu (USC); **Genetics Core Leaders and Key Personnel:** Andrew J. Saykin (Indiana University), Tatiana M. Foroud (Indiana University)**,** Steven Potkin (UC Irvine), Li Shen (Indiana University), Kelley Faber (Indiana University), Sungeun Kim (Indiana University), Kwangsik Nho (Indiana University)**;**

**Initial Concept Planning & Development:** Michael W. Weiner (UC San Francisco), Leon Thal (UC San Diego), Zaven Khachaturian (Prevent Alzheimer's Disease 2020); **Early Project Development:** Zaven Khachaturian (Prevent Alzheimer's Disease 2020), Richard Frank (General Electric), Peter J. Snyder (University of Connecticut), Michael W. Weiner (UC San Francisco), Leon Thal (UC San Diego), Neil Buckholtz (NIA), William Potter (NIMH), Steven Paul (Cornell University), Marilyn Albert (The Johns Hopkins University); **NIA:** John Hsiao (National Institute on Aging/National Institutes of Health) ;

**ADNI Investigators By Site** (FULL ADNI Investigator Lists):
**Oregon Health and Science University:** Jeffrey Kaye, Joseph Quinn, Betty Lind, Raina Carter, Sara Dolen – Past Investigator; **University of Southern California:** Boris A. Gutman, Lon S. Schneider, Sonia Pawluczyk, Mauricio Beccera, Liberty Teodoro, Bryan M. Spann, DO – Past Investigator; **University of California-San Diego:** James Brewer, Helen Vanderswag, Adam Fleisher – Past Investigator; **University of Michigan:** Judith L. Heidebrink, Joanne L. Lord; **Mayo Clinic, Rochester:** Ronald Petersen, Sara S. Mason, Colleen S. Albers, David Knopman, Kris Johnson – Past Investigator; **Baylor College of Medicine:** Rachelle S. Doody, Javier Villanueva-Meyer, Munir Chowdhury, Susan Rountree, Mimi Dang; **Columbia University Medical Center:** Yaakov Stern, Lawrence S. Honig, Karen L. Bell; **Washington University, St. Louis:** Beau Ances, John C. Morris, Maria Carroll, Sue Leon, Erin Householder, Mark A. Mintun – Past Investigator, Stacy Schneider – Past Investigator, Angela Oliver – Past Investigator; **University of Alabama - Birmingham:** Daniel Marson, Randall Griffith, David Clark, David Geldmacher, John Brockington, Erik Roberson; **Mount Sinai School of Medicine:** Hillel Grossman, Effie Mitsis; **Rush University Medical Center:** Leyla deToledo-Morrell, Raj C. Shah; **Wien Center:** Ranjan Duara, Daniel Varon, Maria T. Greig, Peggy Roberts– Past Investigator; **Johns Hopkins University:** Marilyn Albert, Chiadi Onyike, Daniel D'Agostino II, Stephanie Kielb – Past Investigator; **New York University:** James E. Galvin, Dana M. Pogorelec, Brittany Cerbone, Christina A. Michel, Henry Rusinek – Past Investigator, Mony J de Leon – Past Investigator, Lidia Glodzik – Past Investigator, Susan De Santi – Past Investigator; **Duke University Medical Center:** P. Murali Doraiswamy, Jeffrey R. Petrella, Terence Z. Wong; **University of Pennsylvania:** Steven E. Arnold, Jason H. Karlawish, David Wolk; **University of Kentucky:** Charles D. Smith, Greg Jicha, Peter Hardy, Partha Sinha, Elizabeth Oates, Gary Conrad; **University of Pittsburgh:** Oscar L. Lopez, MaryAnn Oakley, Donna M. Simpson; **University of Rochester Medical Center:** Anton P. Porsteinsson, Bonnie S. Goldstein, Kim Martin, Kelly M. Makino – Past Investigator, M. Saleem Ismail – Past Investigator, Connie Brand – Past Investigator; **University of California, Irvine:** Ruth A. Mulnard, Gaby Thai, Catherine Mc-Adams-Ortiz; **University of Texas Southwestern Medical School:** Kyle Womack, Dana Mathews, Mary Quiceno, Ramon Diaz-Arrastia – Past Investigator, Richard King – Past Investigator, Myron Weiner – Past Investigator, Kristen Martin-Cook – Past Investigator, Michael DeVous – Past Investigator;
**Emory University:** Allan I. Levey, James J. Lah, Janet S. Cellar; **University of Kansas, Medical Center:** Jeffrey M. Burns, Heather S. Anderson, Russell H. Swerdlow; **University of California, Los Angeles:** Liana Apostolova, Kathleen Tingus, Ellen Woo, Daniel H.S. Silverman, Po H. Lu – Past Investigator, George Bartzokis – Past Investigator; **Mayo Clinic, Jacksonville:** Neill R Graff-Radford, Francine Parfitt, Tracy Kendall, Heather Johnson – Past Investigator; **Indiana University:** Martin R. Farlow, Ann Marie Hake, Brandy R. Matthews, Scott Herring, Cynthia Hunt; **Yale University School of Medicine:** Christopher H. van Dyck, Richard E. Carson, Martha G. MacAvoy; **McGill Univ., Montreal-Jewish General Hospital:** Howard Chertkow, Howard Bergman, Chris Hosein; **Sunnybrook Health Sciences, Ontario:** Sandra Black, Dr Bojana Stefanovic, Curtis Caldwell; **U.B.C. Clinic for AD & Related Disorders:** Ging-Yuek Robin Hsiung, Howard Feldman, Benita Mudge, Michele Assaly, – Past Investigator; **Cognitive Neurology - St. Joseph's, Ontario:** Andrew Kertesz, John Rogers, Dick Trost;

**Cleveland Clinic Lou Ruvo Center for Brain Health:** Charles Bernick, Donna Munic; **Northwestern University:** Diana Kerwin, Marek-Marsel Mesulam, Kristine Lipowski, Chuang-Kuo Wu – Past Investigator, Nancy Johnson – Past Investigator; **Premiere Research Inst (Palm Beach Neurology):** Carl Sadowsky, Walter Martinez, Teresa Villena; **Georgetown University Medical Center:** Raymond Scott Turner, Kathleen Johnson, Brigid Reynolds; **Brigham and Women's Hospital:** Reisa A. Sperling, Keith A. Johnson, Gad Marshall, Meghan Frey – Past Investigator; **Stanford University:** Jerome Yesavage, Joy L. Taylor, Barton Lane, Allyson Rosen – Past Investigator, Jared Tinklenberg – Past Investigator; **Banner Sun Health Research Institute:** Marwan N. Sabbagh, Christine M. BeldenSandra A. Jacobson, Sherye A. Sirrel; **Boston University:** Neil Kowall, Ronald Killiany, Andrew E. Budson, Alexander Norbash – Past Investigator, Patricia Lynn Johnson – Past Investigator; **Howard University:** Thomas O. Obisesan, Saba Wolday, Joanne Allard; **Case Western Reserve University:** Alan Lerner, Paula Ogrocki, Leon Hudson – Past Investigator; **University of California, Davis – Sacramento:** Evan Fletcher, Owen Carmichael, John Olichney, Charles DeCarli – Past Investigator; **Neurological Care of CNY:** Smita Kittur; **Parkwood Hospital:** Michael Borrie, T-Y Lee, Dr Rob Bartha; **University of Wisconsin:** Sterling Johnson, Sanjay Asthana, Cynthia M. Carlsson; **University of California, Irvine - BIC:** Steven G. Potkin, Adrian Preda, Dana Nguyen; **Banner Alzheimer's Institute:** Pierre Tariot, Adam Fleisher, Stephanie Reeder; **Dent Neurologic Institute:** Vernice Bates, Horacio Capote, Michelle Rainka; **Ohio State University:** Douglas W. Scharre, Maria Kataki, Anahita Adeli; **Albany Medical College:** Earl A. Zimmerman, Dzintra Celmins, Alice D. Brown; **Hartford Hospital, Olin Neuropsychiatry Research Center:** Godfrey D. Pearlson, Karen Blank, Karen Anderson; **Dartmouth-Hitchcock Medical Center:** Robert B. Santulli, Tamar J. Kitzmiller, Eben S. Schwartz – Past Investigator; **Wake Forest University Health Sciences:** Kaycee M. Sink, Jeff D. Williamson, Pradeep Garg, Franklin Watkins – Past Investigator; **Rhode Island Hospital:** Brian R. Ott, Henry Querfurth, Geoffrey Tremont; **Butler Hospital:** Stephen Salloway, Paul Malloy, Stephen Correia; **UC San Francisco:** Howard J. Rosen, Bruce L. Miller; **Medical University South Carolina:** Jacobo Mintzer, Kenneth Spicer, David Bachman; **St. Joseph’s Health Care:** Elizabether Finger, Stephen Pasternak, Irina Rachinsky, John Rogers, Andrew Kertesz – Past Investigator, Dick Drost – Past Investigator; **Nathan Kline Institute:** Nunzio Pomara, Raymundo Hernando, Antero Sarrael; **University of Iowa College of Medicine:** Susan K. Schultz, Laura L. Boles Ponto, Hyungsub Shim, Karen Elizabeth Smith; **Cornell University:** Norman Relkin, Gloria Chaing, Lisa Raudin; **University of South Florida: USF Health Byrd Alzheimer’s Institute:** Amanda Smith, Kristin Fargher, Balebail Ashok Raj.

**ADNI Methods:**

Data used in the preparation of this article were obtained from the Alzheimer’s Disease Neuroimaging Initiative (ADNI) database (adni.loni.usc.edu). The ADNI was launched in 2003 by the National Institute on Aging (NIA), the National Institute of Biomedical Imaging and Bioengineering (NIBIB), the Food and Drug Administration (FDA), private pharmaceutical companies and non-profit organizations, as a $60 million, 5-year public-private partnership. The primary goal of ADNI has been to test whether serial magnetic resonance imaging (MRI), positron emission tomography (PET), other biological markers, and clinical and neuropsychological assessment can be combined to measure the progression of mild cognitive impairment (MCI) and early Alzheimer’s disease (AD). Determination of sensitive and specific markers of very early AD progression is intended to aid researchers and clinicians to develop new treatments and monitor their effectiveness, as well as lessen the time and cost of clinical trials.

The Principal Investigator of this initiative is Michael W. Weiner, MD, VA Medical Center and

University of California – San Francisco. ADNI is the result of efforts of many co-investigators from a broad range of academic institutions and private corporations, and subjects have been recruited from over 50 sites across the U.S. and Canada. The initial goal of ADNI was to recruit 800 subjects but ADNI has been followed by ADNI-GO and ADNI-2. To date these three protocols have recruited over 1500 adults, ages 55 to 90, to participate in the research, consisting of cognitively normal older individuals, people with early or late MCI, and people with early AD. The follow up duration of each group is specified in the protocols for ADNI-1, ADNI-2 and ADNI-GO. Subjects originally recruited for ADNI-1 and ADNI-GO had the option to be followed in ADNI-2. For up-to-date information, see www.adni-info.org.

**The following authors are included under the CHARGE Consortium:**

Najaf Amin (Erasmus University Medical Center, Genetic Epidemiology Unit, Department of Epidemiology and Biostatistics), Diane Becker (General internal Medicine, Johns Hopkins School of Medicine, Baltimore, USA), Alexa Beiser (Department of Biostatistics, Boston University School of Public Health, Boston, MA; Framingham Heart Study, Framingham, MA), Stéphanie Debette (INSERM U897, University of Bordeaux, France; Bordeaux University Hospital; Department of Neurology, Lariboisière Hospital, Paris, France; Department of Neurology, Boston University School of Medicine, Boston, USA), Anita DeStefano (Department of Biostatistics, Boston University School of Public Health, Boston, MA; 2) Framingham Heart Study, Framingham, MA), Edith Hofer (Department of Neurology, Clinical Division of Neurogeriatrics, Institute of Medical Informatics, Statistics and Documentation, Medical University Graz), Albert Hofman (Department of Epidemiology, Erasmus University Medical Center, Rotterdam, The Netherlands), Wiro J. Niessen (Department of Medical Informatics Erasmus University Medical Center, Rotterdam, The Netherlands; (2) Faculty of Applied Sciences, Delft University, The Netherlands), Stephan Seiler (Department of Neurology, Clinical Division of Neurogeriatrics, Medical University Graz), Albert Smith (Icelandic Heart Association), Christophe Tzourio (INSERM U897, University of Bordeaux, France; Bordeaux University Hospital and CIC-EC7 ISPED), Dhananjay Vaidya (General Internal Medicine, Johns Hopkins School of Medicine, Baltimore, USA), Meike W. Vernooij (Departments of Epidemiology and Radiology, Erasmus University Medical Center, Rotterdam, The Netherlands)

**The following authors are included under the EPIGEN Consortium:**
David B. Goldstein (The Centre for Genomics and Population Genetics, Duke University Institute for Genome Sciences and Policy, Durham, North Carolina, USA), Erin L. Heinzen (The Centre for Genomics and Population Genetics, Duke University Institute for Genome Sciences and Policy, Durham, North Carolina, USA), Kevin Shianna (The Centre for Genomics and Population Genetics, Duke University Institute for Genome Sciences and Policy, Durham, North Carolina, USA), Rodney Radtke (Department of Medicine, Duke University Medical Center, Durham, North Carolina, USA) and Ruth Ottmann (Departments of Epidemiology, Neurology, and the G.H. Sergievsky Center, Columbia University, New York, NY).

**The following authors are included under the IMAGEN Consortium:** Lisa Albrecht (Charité), Chris Andrew (IoP), Mercedes Arroyo (Cambridge University), Eric Artiges (INSERM), Semiha Aydin (PTB), Christine Bach (Central Institute of Mental Health), Tobias Banaschewski (Central Institute of Mental Health), Alexis Barbot (Commissariat à l'Energie Atomique), Gareth Barker (IoP), Nathalie Boddaert (INSERM), Arun Bokde (Trinity College Dublin), Zuleima Bricaud (INSERM), Uli Bromberg (University of Hamburg), Ruediger Bruehl (PTB), Christian Büchel (University of Hamburg), Arnaud Cachia (INSERM), Anna Cattrell (IoP), Patricia Conrod (IoP), Patrick Constant (PERTIMM), Hans Crombag (University of Sussex), Katharina Czech (Charité), Jeffrey Dalley (Cambridge University), Benjamin Decideur (Commissariat à l'Energie Atomique),

Sylvane Desrivieres (IoP), Tahmine Fadai (University of Hamburg), Herta Flor (Central Institute of Mental Health), Vincent Frouin (Commissariat à l'Energie Atomique), Birgit Fuchs (GABO:milliarium mbH & Co. KG), Jürgen Gallinat (Charité), Hugh Garavan (Trinity College Dublin), Fanny Gollier Briand (INSERM), Penny Gowland (University of Nottingham), Kay Head (University of Nottingham), Bert Heinrichs (Deutsches Referenzzentrum für Ethik), Andreas Heinz (Charité), Nadja Heym (University of Nottingham), Thomas Hübner (Technische Universität Dresden), Albrecht Ihlenfeld (PTB), James Ireland (Delosis), Bernd Ittermann (PTB), Nikolay Ivanov (Charité), Tianye Jia (IoP), Jennifer Jones (Trinity College Dublin), Arno Klaassen (Scito), Christophe Lalanne (Commissariat à l'Energie Atomique), Mark Lathrop (CNG), Dirk Lanzerath (Deutsches Referenzzentrum für Ethik), Hervé Lemaitre (INSERM), Katharina Lüdemann (Charité), Christine Macare (IoP), Catherine Mallik (IoP), Jean-François Mangin (INSERM), Karl Mann (Central Institute of Mental Health), Adam Mar (Cambridge University), Jean-Luc Martinot (INSERM), Jessica Massicotte (INSERM), Eva Mennigen (Technische Universität Dresden ), Fabiana Mesquita de Carvahlo (IoP), Xavier Mignon (PERTIMM), Ruben Miranda (INSERM), Kathrin Müller (Technische Universität Dresden), Frauke Nees (Central Institute of Mental Health), Charlotte Nymberg (IoP), Marie-Laure Paillere (INSERM), Tomas Paus (University of Toronto), Zdenka Pausova (University of Toronto), Yolanda Pena-Oliver (University of Sussex), Jean-Baptiste Poline (Commissariat à l'Energie Atomique), Luise Poustka (Central Institute of Mental Health), Michael Rapp (Charité), Laurence Reed (IoP), Gabriel Robert (IoP), Jan Reuter (Charité), Marcella Rietschel (Central Institute of Mental Health), Stephan Ripke (Technische Universität Dresden), Tamzin Ripley (University of Sussex), Trevor Robbins (Cambridge University), Sarah Rodehacke (Technische Universität Dresden ), John Rogers (Delosis), Alexander Romanowski (Charité), Barbara Ruggeri (IoP), Christina Schilling (Charité), Christine Schmäl (Central Institute of Mental Health), Dirk Schmidt (Technische Universität Dresden), Sophia Schneider (University of Hamburg), Markus Schroeder (Tembit), Florian Schubert (PTB), Yannick Schwartz (Commissariat à l'Energie Atomique), Michael Smolka (Technische Universität Dresden), Wolfgang Sommer (Central Institute of Mental Health), Rainer Spanagel (Central Institute of Mental Health), Claudia Speiser (GABO:milliarium mbH & Co. KG), Tade Spranger (Deutsches Referenzzentrum für Ethik / Institut of Science and Ethics), Alicia Stedman (University of Nottingham), Sabina Steiner (Central Institute of Mental Health), Dai Stephens (University of Sussex), Nicole Strache (Charité), Andreas Ströhle (Charité), Maren Struve (Central Institute of Mental Health), Naresh Subramaniam (Cambridge University), David Theobald (Cambridge University), Lauren Topper (IoP), Sabine Vollstaedt-Klein (Central Institute of Mental Health), Bernadeta Walaszek (PTB), Henrik Walter (Charité), Katharina Weiß (Charité), Helen Werts (IoP), Robert Whelan (Trinity College Dublin), Steve Williams (IoP), Juliana Yacubian (University of Hamburg), Veronika Ziesch (Technische Universität Dresden ), Monica Zilbovicius (INSERM), C Peng Wong (IoP), Steven Lubbe (IoP), Lourdes Martinez-Medina (IoP), Agnes Kepa (IoP), Alinda Fernandes (IoP), Amir Tahmasebi (University of Toronto)

**The following authors are included under the MCIC:** Randy L. Gollub (Massachusetts General Hospital), Jody M. Shoemaker (The Mind Research Network), Margaret D. King (The Mind Research Network), Tonya White (Erasmus Medical Centre), Stefan Ehrlich (University of Technology – Dresden), Scott R. Sponheim (University of Minnesota), Vincent P. Clark (The Mind Research Network), Jessica A. Turner (The Mind Research Network), Bryon A. Mueller (University of Minnesota), Vince Magnotta (University of Iowa), Daniel O'Leary (University of Iowa), Beng C. Ho (University of Iowa), Stefan Brauns (Charité University Medicine), Dara S. Manoach (Massachusetts General Hospital), Larry Seidman (Beth Israel Deaconess Medical Center), Juan R. Bustillo (University of New Mexico), John Lauriello (University of Missouri), Jeremy Bockholt (University of Iowa), Kelvin O. Lim (University of Minnesota), Bruce R. Rosen (Massachusetts General Hospital), S. Charles Schulz (University of Minnesota), Vince D.

Calhoun (The Mind Research Network), Nancy C. Andreasen (University of Iowa).

**The following authors are included under the SYS Consortium:** Michal Abrahamowicz (McGill University), Daniel Gaudet (University of Montreal), Gabriel Leonard (McGill University), Michel Perron (University of Quebec in Chicoutimi), Louis Richer (University of Quebec in Chicoutimi), Jean Seguin (University of Montreal), Suzanne Veillette (CEGEP Jonquiere).

## Supplementary Note 2: Acknowledgements

**CHARGE**: Infrastructure for the CHARGE Consortium is supported in part by the National Heart, Lung, and Blood Institute grant HL105756 and for the neuroCHARGE phenotype working group through the National Institute on Aging grant AG033193.

**ENIGMA**: ENIGMA was supported in part by a Consortium grant (U54 EB020403 to PMT) from the NIH Institutes contributing to the Big Data to Knowledge (BD2K) Initiative, including the NIBIB and NCI.

**Cohort-specific acknowledgements:**

**ADNI:** Data collection and sharing for this project was funded by the Alzheimer's Disease Neuroimaging Initiative (ADNI) (National Institutes of Health Grant U01 AG024904) and DOD ADNI (Department of Defense award number W81XWH-12-2-0012). ADNI is funded by the National Institute on Aging, the National Institute of Biomedical Imaging and Bioengineering, and through generous contributions from the following: Alzheimer's Association; Alzheimer's Drug Discovery Foundation; BioClinica, Inc.; Biogen Idec Inc.; Bristol-Myers Squibb Company; Eisai Inc.; Elan Pharmaceuticals, Inc.; Eli Lilly and Company; F. Hoffmann-La Roche Ltd and its affiliated company Genentech, Inc.; GE Healthcare; Innogenetics, N.V.; IXICO Ltd.; Janssen Alzheimer Immunotherapy Research & Development, LLC.; Johnson & Johnson Pharmaceutical Research & Development LLC.; Medpace, Inc.; Merck & Co., Inc.; Meso Scale Diagnostics, LLC.; NeuroRx Research; Novartis Pharmaceuticals Corporation; Pfizer Inc.; Piramal Imaging; Servier; Synarc Inc.; and Takeda Pharmaceutical Company. The Canadian Institutes of Health Research is providing funds to support ADNI clinical sites in Canada. Private sector contributions are facilitated by the Foundation for the National Institutes of Health (www.fnih.org). The grantee organization is the Northern California Institute for Research and Education, and the study is coordinated by the Alzheimer's Disease Cooperative Study at the University of California, San Diego. ADNI data are disseminated by the Laboratory of Neuro Imaging at the University of Southern California.

**Age, Gene/Environment Susceptibility-Reykjavik Study (AGES-Reykjavik)**: This study has been funded by NIH contract N01-AG-1-2100, the NIA Intramural Research Program, Hjartavernd (the Icelandic Heart Association), and the Althingi (the Icelandic Parliament). The study is approved by the Icelandic National Bioethics Committee, VSN: 00–063. The researchers are indebted to the participants for their willingness to participate in the study.

**ANM:** AddNeuroMed was funded through the EU FP6 programme. HS: Academy of Finland, Research Council for Health, 258081, UEFBrain, University of Eastern Finland, VTR funding Kuopio University Hospital.

**Atherosclerosis Risk In Communities Study (ARIC):** The Atherosclerosis Risk in Communities study was performed as a collaborative study supported by National Heart, Lung, and Blood Institute (NHLBI) contracts (HHSN268201100005C, HSN268201100006C, HSN268201100007C, HHSN268201100008C, HHSN268201100009C, HHSN268201100010C, HHSN268201100011C, and HHSN268201100012C), R01HL70825, R01HL087641, R01HL59367, and R01HL086694; National Human Genome Research Institute contract U01HG004402; and National Institutes of Health (NIH) contract HHSN268200625226C. Infrastructure was partly supported by grant No. UL1RR025005, a component of the NIH and NIH Roadmap for Medical Research. This project was partially supported by National Institutes of Health R01 grants HL084099 and NS087541 to MF.

**Austrian Stroke Prevention Study (ASPS)**: The research reported in this article was funded by the Austrian Science Fond (FWF) grant number P20545-P05 and P13180. The Medical University of Graz supports the databank of the ASPS. The authors thank the staff and the participants of the ASPS for their valuable contributions. We thank Birgit Reinhart for her long-term administrative commitment and Ing Johann Semmler for the technical assistance at creating the DNA-bank.

**Austrian Stroke Prevention Study Family (ASPS-Fam)**: The ASPS-Fam is funded by the Austrian Science Fund (FWF) project I904, the Medical University of Graz and the Steiermärkische Krankenanstalten Gesellschaft.

**BETULA:** This sample collection was supported by a Wallenberg Scholar grant from the Knut and Alice Wallenberg (KAW) foundation and a grant from Torsten and Ragnar Söderbergs Foundation to Lars Nyberg. Stephanie le Hellard was supported by a grant from HelseVest RHF (Grant 911554).

**Bipolar Family Study:** The Bipolar Family Study wishes to thank the Scottish Mental Health Research Network for research assistant support, the Brain Research Imaging Centre Edinburgh, a center in the Scottish Funding Council Scottish Imaging Network–A Platform for Scientific Excellence (SINAPSE) Collaboration, for image acquisition and the Wellcome Trust Clinical Research Facility for genotyping. Genotyping was supported by the National Alliance for Research on Schizophrenia and Depression (NARSAD) Independent Investigator Award (to A.M.M.), and data collection was supported by the Health Foundation Clinician Scientist Fellowship. The research leading to these results also receives funding from the European Community's Seventh Framework Programme (FP7/2007– 2013) under grant agreements #602450 (IMAGEMEND) and ongoing support from the Wellcome Trust (Ref 104036/Z/14/Z).

**BIG:** This work makes use of the BIG (Brain Imaging Genetics) database, first established in Nijmegen, The Netherlands, in 2007. This resource is now part of Cognomics (www.cognomics.nl), a joint initiative by researchers of the Donders Centre for Cognitive Neuroimaging, the Human Genetics and Cognitive Neuroscience departments of the Radboud university medical centre and the Max Planck Institute for Psycholinguistics in Nijmegen. The Board of the Cognomics Initiative consists of Barbara Franke, Simon Fisher, Guillen Fernandez, Peter Hagoort, Han Brunner, Jan Buitelaar, Hans van Bokhoven and David Norris. The Cognomics Initiative has received supported from the participating departments and centres and from external grants, i.e. the Biobanking and Biomolecular Resources Research Infrastructure (Netherlands) (BBMRI-NL), the Hersenstichting Nederland, and the Netherlands Organisation for Scientific Research (NWO). The research leading to these results also receives funding from the NWO Gravitation grant 'Language in Interaction', the European Community's Seventh Framework Programme (FP7/2007– 2013) under grant agreements n° 602450 (IMAGEMEND), n° 278948 (TACTICS), and n°602805 (Aggressotype) as well as from the European Community's Horizon 2020 programme under grant agreement n° 643051 (MiND) and from ERC-2010-AdG 268800-NEUROSCHEMA. In addition, the work was supported by a grant for the ENIGMA Consortium (grant number U54 EB020403) from the BD2K Initiative of a cross-NIH partnership. We wish to thank all persons who kindly participated in the BIG research.

**Brain Genomics Superstruct Project (GSP):** Data were provided [in part] by the Brain Genomics Superstruct Project of Harvard University and the Massachusetts General Hospital,

with support from the Center for Brain Science Neuroinformatics Research Group, the Athinoula A. Martinos Center for Biomedical Imaging, and the Center for Human Genetic Research. 20 individual investigators at Harvard and MGH generously contributed data to GSP. P.H.L is supported by NIH K99 MH101367. JWS is supported in part by NIMH K24MH094614 and as a Tepper Family MGH Research Scholar

**Cardiovascular Health Study:** This CHS research was supported by NHLBI contracts HHSN268201200036C, HHSN268200800007C, N01HC55222, N01HC85079, N01HC85080, N01HC85081, N01HC85082, N01HC85083, N01HC85086, N01HC15103; and NHLBI grants HL080295, HL087652, HL105756, HL103612, and HL120393 with additional contribution from the National Institute of Neurological Disorders and Stroke (NINDS). Additional support was provided through AG023629 and AG15928 from the National Institute on Aging (NIA). A full list of principal CHS investigators and institutions can be found at CHS-NHLBI.org/. The provision of genotyping data was supported in part by the National Center for Advancing Translational Sciences, CTSI grant UL1TR000124, and the National Institute of Diabetes and Digestive and Kidney Disease Diabetes Research Center (DRC) grant DK063491 to the Southern California Diabetes Endocrinology Research Center. The content is solely the responsibility of the authors and does not necessarily represent the official views of the National Institutes of Health.

**Epidemiology of Dementia in Singapore (EDIS)**: The Singapore Malay Eye Study (SiMES) and the Singapore Chinese Eye. Study (SCES) are funded by National Medical Research Council (grants 0796/2003, IRG07nov013, IRG09nov014, STaR/0003/2008 and CG/SERI/2010) and Biomedical Research Council (grants 09/1/35/19/616), Singapore. The Genome Institute of Singapore, Agency for Science, Technology and Research, Singapore provided services for genotyping. The Epidemiology of Dementia in Singapore study is supported by the National Medical Research Council, Singapore (NMRC/CG/NUHS/2010 [Grant no: R-184-006-184-511]). Dr. M.K. Ikram received additional funding from the Singapore Ministry of Health's National Medical Research Council (NMRC/CSA/038/2013).

**EPIGEN:** Work from the London Cohort was supported by research grants from the Wellcome Trust (grant 084730 to S.M.S.), University College London (UCL)/University College London Hospitals (UCLH) NIHR Biomedical Research Centre/Specialist Biomedical Research Centres (CBRC/SBRC) (grant 114 to S.M.S.), the European Union Marie Curie Reintegration (to M. Matarin and S.M.S.), the UK NIHR (08-08-SCC), the Comprehensive Local Research Network (CLRN) Flexibility and Sustainability Funding (FSF) (grant CEL1300 to S.M.S.), The Big Lottery Fund, the Wolfson Trust and the Epilepsy Society. This work was undertaken at UCLH/UCL, which received a proportion of funding from the UK Department of Health's NIHR Biomedical Research Centres funding scheme. Work from the Royal College of Surgeons in Ireland was supported by research grants from the Science Foundation Ireland (Research Frontiers Programme award 08/RFP/GEN1538) and Brainwave–the Irish Epilepsy Association. M. Matarin is funded by Epilepsy Research UK (grant F1206).

**Framingham Heart Study (FHS)**: This work was supported by the National Heart, Lung and Blood Institute's Framingham Heart Study (Contract No. N01-HC-25195) and its contract with Affymetrix, Inc for genotyping services (Contract No. N02-HL-6-4278). This study was also supported by grants from the National Institute of Aging (R01s AG033193, AG08122, AG16495, U0149505), the National Institute of Neurological Disorders and Stroke (R01 NS17950), the National Heart, Lung and Blood Institute (R01 HL093029, HL096917). C.Decarli is supported by Alzheimer's Disease Center (P30 AG 010129).

**GeneSTAR:** was supported by grants from the National Institutes of Health National Institute of

Neurological Disorders and Stroke (R01NS062059), the National Institutes of Health National Heart, Lung, and Blood Institute (U01 HL72518, HL097698) and the National Institutes of Health/National Center for Research Resources (M01-RR000052) to the Johns Hopkins General Clinical Research Center. We would like to thank the participants and families of GeneSTAR and our dedicated staff for all their sacrifices.

**GIG:** The GIG (Genomic Imaging Göttingen) sample was established at the Center for Translational Research in Systems Neuroscience and Psychiatry at Göttingen University. We thank Maria Keil, Esther Diekhof, Tobias Melcher and Ilona Henseler for assistance in MRI data acquisition, and Elisabeth Binder and Holger Mohr for their valuable help with genotyping. We are grateful to all persons who kindly participated in the GIG study.

**Genetics of Brain Structure and Function (GOBS) Study:** We acknowledge the ultimate source of our data, the Mexican American community of San Antonio and surrounding areas. Financial support for this study was provided by grants from the National Institute of Mental Health MH0708143 (Principal Investigator [PI]: DC Glahn), MH078111 (PI: J Blangero), and MH083824 (PI: Glahn & Blangero). Theoretical development of SOLAR is supported by MH59490 (PI: Blangero). This investigation was conducted, in part, in facilities constructed with support from Research Facilities Improvement Program Grant Numbers C06 RR13556 and C06 RR017515 from the National Center for Research Resources, NIH. Some of this work was performed at Texas Biomedical Research Institute where Dr. Blangero began this investigator-initiated competitively publicly funded work. Authors declare no competing financial interests in relation to the described work. AJH is supported through NIMH grant K01MH099232.

**HUBIN:** This study was financed by the Swedish Research Council (K2007-62X-15077-04-1, K2008-62P-20597-01-3. K2010-62X-15078-07-2, K2012-61X-15078-09-3), the regional agreement on medical training and clinical research between Stockholm County Council and the Karolinska Institutet, the Knut and Alice Wallenberg Foundation, and the HUBIN project. Genotyping was performed by the SNP&SEQ Technology Platform in Uppsala. The platform is part of Science for Life Laboratory at Uppsala University and supported as a national infrastructure by the Swedish Research Council.

**HUNT:** The Nord-Trøndelag Health Study (The HUNT Study) is a collaboration between HUNT Research Centre (Faculty of Medicine, Norwegian University of Science and Technology NTNU), Nord-Trøndelag County Council, Central Norway Health Authority, and the Norwegian Institute of Public Health (https://www.ntnu.edu/hunt). The HUNT MRI study and the GWAS analysis in this cohort were supported by grants from the Liaison Committee between the Central Norway Regional Health Authority and the Norwegian University of Science and Technology, and Department of Health and Social Services appointed Norwegian National Advisory Unit for functional MRI.

**IMAGEN:** IMAGEN was supported by the European Union-funded FP6 Integrated Project IMAGEN (Reinforcement- related behaviour in normal brain function and psychopathology) (LSHM-CT- 2007-037286), the FP7 projects IMAGEMEND (602450) and MATRICS (603016), and the Innovative Medicine Initiative Project EU-AIMS (115300-2), the Medical Research Council Programme Grant "Developmental pathways into adolescent substance abuse" (93558), as well as the NIHR-biomedical Research Center "Mental Health". Further support was provided by the Swedish Research Council FORMAS, the German Federal Ministry for Education and Research BMBF (eMED SysAlc 01ZX1311A; Forschungsnetz AERIAL; 1EV0711), the National Institutes of Health, U.S.A. (Axon, Testosterone and Mental Health during Adolescence; MH085772-01A1), Eranet – Neuron grant (AF12-NEUR0008-01 -

WM2NA), MILDECA, Fondation pour la Recherche Médicale, and IDEX Paris - Saclay.

**IMpACT:** This study was funded by a grant from the Brain & Cognition Excellence Program of the Netherlands Organization for Scientific Research (NWO, grant 433-09-229) and in part by the Netherlands Brain Foundation (grant number, 15F07[2]27). B. Franke is supported by a Vici grant from the Netherlands Organisation for Scientific Research (NWO; grant n° 016.130.669). The research leading to these results also receives funding from the European Community's Seventh Framework Programme (FP7/2007– 2013) under grant agreements n° 602450 (IMAGEMEND), n°278948 (TACTICS), and n°602805 (Aggressotype) as well as from the European Community's Horizon 2020 programme under grant agreement n° 643051 (MiND). In addition, the work was supported by a grant for the ENIGMA Consortium (grant number U54 EB020403) from the BD2K Initiative of a cross-NIH partnership.

**LBC1936:** The work was undertaken as part of the Cross Council and University of Edinburgh Centre for Cognitive Ageing and Cognitive Epidemiology (CCACE; http://www.ccace.ed.ac.uk). This work was supported by a Research into Ageing programme grant (to I.J.D.) and the Age UK-funded Disconnected Mind project (http://www.disconnectedmind.ed.ac.uk; to I.J.D. and J.M.W.), with additional funding from the UK Medical Research Council (MRC; to I.J.D., J.M.W. and M.E.B.). The whole genome association part of this study was funded by the Biotechnology and Biological Sciences Research Council (BBSRC; Ref. BB/F019394/1). J.M.W. is supported by the Scottish Funding Council through the SINAPSE Collaboration (http://www.sinapse.ac.uk). CCACE (MRC MR/K026992/1) is funded by the BBSRC and MRC. The image acquisition and analysis was performed at the Brain Research Imaging Centre, University of Edinburgh (http://www.bric.ed.ac.uk).

**Leiden Longevity Study (LLS)**: The Leiden Longevity Study was supported by a grant from the Innovation-Oriented Research Program on Genomics [SenterNovem IGE05007] and the Netherlands Consortium for Healthy Ageing [grant number 050-060-810].

**MCIC:** Data used in the preparation of this work were obtained from the Mind Clinical Imaging Consortium database through the Mind Research Network (www.mrn.org). The MCIC project was supported by the Department of Energy under Award Number DE-FG02-08ER64581. MCIC is the result of efforts of co-investigators from University of Iowa, University of Minnesota, University of New Mexico, Massachusetts General Hospital.

**MooDS:** The establishment of the MooDS sample was funded by the German Federal Ministry of Education and Research (BMBF) through the Integrated Genome Research Network (IG) MooDS (Systematic Investigation of the Molecular Causes of Major Mood Disorders and Schizophrenia; grant 01GS08144 to Markus M. Nöthen and Sven Cichon, grant 01GS08147 to Marcella Rietschel and Andreas Meyer-Lindenberg, grant 01GS08144 to Henrik Walter and grant 01GS08148 to Andreas Heinz),) under the auspices of the National Genome Research Network plus (NGFNplus), and through the Integrated Network IntegraMent (Integrated Understanding of Causes and Mechanisms in Mental Disorders), under the auspices of the e:Med Programme (grant 01ZX1314A to Markus M. Nöthen, grant 01ZX1314C to Henrik Walter, grant 01ZX1314G to Marcella Rietschel).

**MPIP:** The MPIP Munich Morphometry Sample comprises images acquired as part of the Munich Antidepressant Response Signature Study and the Recurrent Unipolar Depression (RUD) Case-Control study performed at the MPIP, and control subjects acquired at the Ludwig-Maximilians-University, Munich, Department of Psychiatry. We thank Eva Meisenzahl and Dan Rujescu for providing MRI and genetical data for inclusion into the MPIP Munich Morphometry

sample. We wish to acknowledge Anna Olynyik and radiographers Rosa Schirmer, Elke Schreiter, Reinhold Borschke and Ines Eidner for image acquisition and data preparation. We thank Dorothee P. Auer for local study management in the initial phase of the RUD study. We are grateful to GlaxoSmithKline for providing the genotypes of the Recurrent Unipolar Depression Case-Control Sample. We thank the staff of the Center of Applied Genotyping (CAGT) for generating the genotypes of the MARS cohort. The study is supported by a grant of the Exzellenz-Stiftung of the Max Planck Society. This work has also been funded by the Federal Ministry of Education and Research (BMBF) in the framework of the National Genome Research Network (NGFN), FKZ 01GS0481.

**NCNG:** this sample collection was supported by grants from the Bergen Research Foundation and the University of Bergen, the Dr Einar Martens Fund, the K.G. Jebsen Foundation, the Research Council of Norway, to SLH, VMS and TE.

**NESDA:** Funding was obtained from the Netherlands Organization for Scientific Research (Geestkracht program grant 10-000-1002); the Center for Medical Systems Biology (CSMB, NWO Genomics), Biobanking and Biomolecular Resources Research Infrastructure (BBMRI-NL), VU University's Institutes for Health and Care Research (EMGO+) and Neuroscience Campus Amsterdam, University Medical Center Groningen, Leiden University Medical Center, National Institutes of Health (NIH, R01D0042157-01A, MH081802, Grand Opportunity grants 1RC2 MH089951 and 1RC2 MH089995). Part of the genotyping and analyses were funded by the Genetic Association Information Network (GAIN) of the Foundation for the National Institutes of Health.Computing was supported by BiG Grid, the Dutch e-Science Grid, which is financially supported by NWO.

**NeuroIMAGE:** The NeuroIMAGE was supported by NIH Grant R01MH62873 (to Stephen V. Faraone), NWO Large Investment Grant 1750102007010 (to Jan Buitelaar), the European Community's Seventh Framework Programme (FP7/2007– 2013) under grant agreements n° 602450 (IMAGEMEND), n°278948 (TACTICS) and by grants from Radboud University Nijmegen Medical Center, University Medical Center Groningen and Accare, and VU University Amsterdam.

**NIMH-IRP:** Supported in part by the NIMH Intramural Research Program (ZIAMH002810; Z01MH002792; Z01MH002790).

**NOMAS:** Northern Manhattan Study (NOMAS) is supported by the NINDS (grants R37 NS29993 and K02 NS 059729). Genome-wide data were supported by the Evelyn F. McKnight Brain Institute.

**NTR-Adults and Brainscale:** We would like to thank all twin participants from the Netherlands Twin Register. The NTR-adult and Brainscale studies were supported by the Netherlands Organization for Scientific Research NWO [MW904-61-193 (E.d.G & D.B), MaGW-nr: 400-07-080 (D. v't E.), MagW 480-04-004 (D.B), (51.02.060 (H.H.), 668.772 (D.B. & H.H.); NWO/SPI 56-464-14192 (D.B.), the European Research Council (ERC-230374) (D.B.), High Potential Grant Utrecht University (H.H.), NWO Brain and Cognition 433-09-220 (H.H.) and the Neuroscience Campus Amsterdam (NCA).

**NUIG-TCD:** Galway (NUI Galway) The Health Research Board, the National University of Ireland Galway Millennium Fund, and the Welcome Trust; (b) For Dublin (Trinity College Dublin) these are: Science Foundation Ireland, The Health Research Board, and The Irish Research Council.

**Older Australian Twins Study (OATS)**: We would like to acknowledge and thank the OATS participants, their supporters and respective Research Teams. This work was supported by a number of sources. OATS is supported by the NHMRC/Australian Research Council Strategic Award 401162 and NHMRC Project Grant 1045325 to P. Sachdev and colleagues. OATS was facilitated through access to the Australian Twin Registry, a national research resource supported by the NHMRC Enabling Grant 310667, administered by the University of Melbourne. DNA was extracted by Genetic Repositories Australia, an Enabling Facility supported by the NHMRC Grant 401184. OATS genotyping was partly funded by a Commonwealth Scientific and Industrial Research Organisation Flagship Collaboration Fund Grant. Henry Brodaty is supported by the Australian Government funded Dementia Collaborative Research Centre (DCRC), UNSW. Nicola Armstrong was supported by the NHMRC Project Grant 525453 and Karen Mather is supported by an Alzheimer's Australia Dementia Research Foundation Postdoctoral Fellowship and the NHMRC Capacity Building Grant 568940.

**Osaka:** This study was supported, in part, by research grants from the Japanese Ministry of Health, Labor and Welfare; the Japanese Ministry of Education, Culture, Sports, Science and Technology (MEXT) KAKENHI and Scientific Research on Innovative Areas (Comprehensive Brain Science Network)]; and the Brain Sciences Project of the Center for Novel Science Initiatives (CNSI), the National Institutes of Natural Sciences (NINS), and the Brain Mapping by Integrated Neurotechnologies for Disease Studies (Brain/MINDS) from Japan Agency for Medical Research and development, AMED.

**PAFIP:** The PAFIP study was supported by Instituto de Salud Carlos III, FIS00/3095, 01/3129, PI020499, PI060507, PI10/00183, PI14/00639, the SENY Fundació Research Grant CI 2005-0308007, and the Fundación Marqués de Valdecilla API07/011. PAFIP wish to acknowledge WTCCC2 (Wellcome Trust Case Control Consortium 2) for DNA Genotyping, Valdecilla Biobank for providing the biological samples and associated data included in this study and Idival Neuroimaging Unit for its help in the technical execution of this work. Diana Tordesillas-Gutiérrez is funded by a contract from the Carlos III Health Institute (CA12/00312).

**PROSPER**: The PROSPER study was supported by an investigator initiated grant obtained from Bristol-Myers Squibb. Prof. Dr. J. W. Jukema is an Established Clinical Investigator of the Netherlands Heart Foundation (grant 2001 D 032). Support for genotyping was provided by the seventh framework program of the European commission (grant 223004) and by the Netherlands Genomics Initiative (Netherlands Consortium for Healthy Aging grant 050-060-810).

**QTIM:** DPH, NJ, CRKC, and PMT are supported, in part, by NIH grants R01 NS080655, R01AG040060, R01 EB008432, R01 MH097268, U01 AG024904, R01 MH085667, R01 MH089722, P41 EB015922, and R01 MH094343. RKW is supported by National Science Foundation (BCS-1229450). JLS was supported by the NIMH (K99MH102357) and Autism Speaks. SEM and GZ are supported by Future Fellowships (FT110100548, FT0991634) from the Australian Research Council, and GWM is supported by a National Health and Medical Research Council (NHMRC), Australia, Fellowship (619667). The QTIM study is supported by grants from NIH (R01 HD050735) and the NHMRC (389875, 486682, 1009064). We thank the twins and siblings for their participation, Marlene Grace and Ann Eldridge for twin recruitment, Aiman Al Najjar and other radiographers for scanning, Kerrie McAloney and Daniel Park for research support, and Anjali Henders and staff for DNA sample processing and preparation.

**Rotterdam Study (RSI, RSII, RSIII, RSIx):** The Rotterdam Study is supported by the Erasmus

Medical Center and Erasmus University Rotterdam, the NWO, ZonMw, the Research Institute for Diseases in the Elderly (RIDE), The Netherlands Genomics Initiative, the Ministry of Education, Culture and Science, the Ministry of Health, Welfare and Sports, the European Commission (DG XII), and the Municipality of Rotterdam. M.A.I. is supported by ZonMW grant number 916.13.054.

**ROSMAP1 and ROSMAP2**: The clinical, genomic, and neuroimaging data for the Religious Orders Study and the Rush Memory and Aging Project was funded by NIH grants P30AG10161, RF1AG15819, R01AG17917, R01AG30146, R01AG40039, and the Translational Genomics Research Institute.

**SHIP:** The Study of Health in Pomerania (SHIP) is supported by the German Federal Ministry of Education and Research (grants 01ZZ9603, 01ZZ0103 and 01ZZ0403) and the German Research Foundation (DFG; GR 1912/5-1). Genome-wide data and MRI scans were supported by a joint grant from Siemens Healthcare, Erlangen, Germany, and the Federal State of Mecklenburg–West Pomerania.

**SHIP-TREND-0:** This cohort is part of the Community Medicine Research net (CMR) of the University of Greifswald, which is funded by the German Federal Ministry of Education and Research and the German Ministry of Cultural Affairs, as well as by the Social Ministry of the Federal State of Mecklenburg–West Pomerania. CMR encompasses several research projects that share data from the population-based Study of Health in Pomerania (SHIP; see URLs). MRI scans were supported by a joint grant from Siemens Healthcare, Erlangen, Germany, and the Federal State of Mecklenburg–West Pomerania. The SHIP authors are grateful to Mario Stanke for the opportunity to use his server cluster for SNP imputation as well as to Holger Prokisch and Thomas Meitinger (HelmholtzZentrum München) for genotyping the SHIP-TREND cohort which was supported by the Federal Ministry of Education and Research (grant 03ZIK012). We thank all staff members and participants of the SHIP studies, as well as all of the genotyping staff for generating the SHIP SNP data set. D. J. is supported by a scholarship from the Gerhard-Domagk programme of the University Medicine Greifswald.

**Sydney Memory and Ageing Study (Sydney MAS)**: We would like to thank the Sydney MAS participants, their supporters and respective Research Teams. Sydney MAS was supported by the Australian National Health and Medical Research Council (NHMRC) Program Grants 350833 and 568969 to P Sachdev, H Brodaty and G Andrews. DNA was extracted by Genetic Repositories Australia, an Enabling Facility supported by the NHMRC Grant 401184. Henry Brodaty is supported by the Australian Government funded Dementia Collaborative Research Centre (DCRC), UNSW. Nicola Armstrong was supported by the NHMRC Project Grant 525453 and Karen Mather is supported by an Alzheimer's Australia Dementia Research Foundation Postdoctoral Fellowship. Both Simone Reppermund and Karen Mather are supported by the NHMRC Capacity Building Grant 568940.

**SYS:** The Saguenay Youth Study project is funded by the Canadian Institutes of Health Research (TP, ZP), Heart and Stroke Foundation of Quebec (ZP), and the Canadian Foundation for Innovation (ZP). TP is supported by the Tanenbaum Chair in Population Neuroscience at the Rotman Research Institute, University of Toronto.

**TOP:** The study was supported by the Research Council of Norway (#213837, #223273, #229129), South-East Norway Health Authority (#2013-123), EU FP7 (#602450) and KG Jebsen Foundation. The research leading to these results has received funding from the European Community's Seventh Framework Programme (FP7/2007-2013) under grant

agreement n° 602450 (IMAGEMEND).

**Three City Study (3C-Dijon):** We thank the staff and the participants of the 3C Study for their important contributions. The 3C Study is conducted under a partnership agreement between the Institut National de la Santé et de la Recherche Médicale (INSERM), the Victor Segalen–Bordeaux II University, and Sanofi-Aventis. The Fondation pour la Recherche Médicale funded the preparation and initiation of the study. The 3C Study is also supported by the Caisse Nationale Maladie des Travailleurs Salariés, Direction Générale de la Santé, Mutuelle Générale de l'Education Nationale (MGEN), Institut de la Longévité, Conseils Régionaux of Aquitaine and Bourgogne, Fondation de France, and Ministry of Research–INSERM Programme "Cohortes et collections de données biologiques." Lille Génopôle received an unconditional grant from Eisai. We thank A. Boland (Centre National de Génotypage) for her technical help in preparing the DNA samples for analyses. This work was supported by the National Foundation for Alzheimer's Disease and Related Disorders, the Institut Pasteur de Lille and the Centre National de Génotypage. Ganesh Chauhan is supported by a grant from the Fondation Leducq. We also thank Fabrice Crivello and Carole Dufouil for their support.

**UCLA_NL_BP:** Data collection and genotyping was made possible with (NIH/NIMH) R01 MH090553 to R.A.O.

**UKBEC:** This work was supported by the UK Medical Research Council (MRC) through the MRC Sudden Death Brain Bank (C.S.), a Project Grant (G0901254 to J.H. and M.E.W.) and Training Fellowship (G0802462 to M.R.). D.T. was supported by the King Faisal Specialist Hospital and Research Centre, Saudi Arabia.

**UMCU:** This work was supported by 917.46.370 (H.H.) and 908-02-123 (H.H.) from the Netherlands Organisation for Health Research and Development ZonMW.

**University of Warwick:** Thomas E. Nichols is supported by the Wellcome Trust.

**WHICAP:** This study was supported by R01-AG034189, P01-AG007232 , R01-AG037212

**Supplementary Note: Author Contributions**

**The following authors wrote and edited the first draft:** Derrek P Hibar, Hieab HH Adams, Neda Jahanshad, Ganesh Chauhan, Jason L Stein, Edith Hofer, Miguel E Renteria, Joshua C Bis, Alejandro Arias-Vasquez, M Kamran Ikram, Sylvane Desrivières, Meike W Vernooij, Nicholas G Martin, Cornelia M Van Duijn, Margaret J Wright, WT Longstreth Jr, Gunter Schumann, Hans J Grabe, Barbara Franke, Lenore J Launer, Sarah E Medland, Sudha Seshadri, Paul M Thompson, M Arfan Ikram; **performed imaging and genetic analyses:** Derrek P Hibar, Hieab HH Adams, Neda Jahanshad, Ganesh Chauhan, Jason L Stein, Edith Hofer, Miguel E Renteria, Joshua C Bis, Alejandro Arias-Vasquez, M Kamran Ikram, Sylvane Desrivières, Meike W Vernooij, Lucija Abramovic, Saud Alhusaini, Najaf Amin, Micael Andersson, Konstantinos Arfanakis, Benjamin S Aribisala, Nicola J Armstrong, Lavinia Athanasiu, Tomas Axelsson, Ashley H Beecham, Alexa Beiser, Manon Bernard, Susan H Blanton, Marc M Bohlken, Marco P Boks, Janita Bralten, Adam M Brickman, Owen Carmichael, M Mallar Chakravarty, Qiang Chen, Christopher RK Ching, Vincent Chouraki, Fabrice Crivello, Gabriel Cuellar-Partida, Anouk Den Braber, Nhat Trung Doan, Stefan Ehrlich, Sudheer Giddaluru, Aaron L Goldman, Rebecca F Gottesman, Oliver Grimm, Michael E Griswold, Tulio Guadalupe, Boris A Gutman, Johanna Hass, Unn K Haukvik, David Höhn, Avram J Holmes, Martine Hoogman, Deborah Janowitz, Tianye Jia, Kjetil N Jørgensen, Nazanin Karbalai, Dalia Kasperaviciute, Sungeun Kim, Marieke Klein, Bernd Krämer, Phil H Lee, David CM Liewald, Lorna M Lopez, Michelle Luciano, Christine Macare, Andre Marquand, Mar Matarin, Karen A Mather, Manuel Mattheisen, David R McKay, Yuri Milaneschi, Susanna Muñoz Maniega, Kwangsik Nho, Allison C Nugent, Paul Nyquist, Loes M Olde Loohuis, Jaap Oosterlaan, Martina Papmeyer, Lukas Pirpamer, Benno Pütz, Adaikalavan Ramasamy, Jennifer S Richards, Shannon L Risacher, Roberto Roiz-Santiañez, Nanda Rommelse, Stefan Ropele, Emma J Rose, Natalie A Royle, Tatjana Rundek, Philipp G Sämann, Claudia L Satizabal, Lianne Schmaal, Andrew J Schork, Li Shen, Jean Shin, Elena Shumskaya, Albert V. Smith, Emma Sprooten, Lachlan T Strike, Alexander Teumer, Diana Tordesillas-Gutierrez, Roberto Toro, Daniah Trabzuni, Stella Trompet, Dhananjay Vaidya, Jeroen Van der Grond, Sven J Van der Lee, Dennis van der Meer, Marjolein MJ Van Donkelaar, Kristel R Van Eijk, Theo GM van Erp, Daan van Rooij, Esther Walton, Lars T Westlye, Christopher D Whelan, Beverly G Windham, Anderson M Winkler, Katharina Wittfeld, Girma Woldehawariat, Christiane Wolf, Thomas Wolfers, Lisa R Yanek, Jingyun Yang, Alex Zijdenbos, Marcel P Zwiers; **local study management and oversight:** Ingrid Agartz, Laura Almasy, David Ames, Philippe Amouyel, Ole A Andreassen, Sampath Arepalli, Amelia A Assareh, Sandra Barral, Mark E Bastin, Diane M Becker, James T Becker, David A Bennett, John Blangero, Hans van Bokhoven, Dorret I Boomsma, Henry Brodaty, Rachel M Brouwer, Han G Brunner, Randy L Buckner, Jan K Buitelaar, Kazima B Bulayeva, Wiepke Cahn, Vince D Calhoun, Dara M Cannon, Gianpiero L Cavalleri, Ching-Yu Cheng, Sven Cichon, Mark R Cookson, Aiden Corvin, Benedicto Crespo-Facorro, Joanne E Curran, Michael Czisch, Anders M Dale, Gareth E Davies, Anton JM De Craen, Eco JC De Geus, Philip L De Jager, Greig I De Zubicaray, Ian J Deary, Stéphanie Debette, Charles DeCarli, Norman Delanty, Chantal Depondt, Anita DeStefano, Allissa Dillman, Srdjan Djurovic, Gary Donohoe, Wayne C Drevets, Ravi Duggirala, Thomas D Dyer, Christian Enzinger, Susanne Erk, Thomas Espeseth, Iryna O Fedko, Guillén Fernández, Luigi Ferrucci,

Simon E Fisher, Debra A Fleischman, Ian Ford, Myriam Fornage, Tatiana M Foroud, Peter T Fox, Clyde Francks, Masaki Fukunaga, J Raphael Gibbs, David C Glahn, Randy L Gollub, Harald HH Göring, Robert C Green, Oliver Gruber, Vilmunder Gudnason, Sebastian Guelfi, Asta K. Håberg, Narelle K Hansell, John Hardy, Catharina A Hartman, Ryota Hashimoto, Katrin Hegenscheid, Andreas Heinz, Stephanie Le Hellard, Dena G Hernandez, Dirk J Heslenfeld, Beng-Choon Ho, Pieter J Hoekstra, Wolfgang Hoffmann, Albert Hofman, Florian Holsboer, Georg Homuth, Norbert Hosten, Jouke-Jan Hottenga, Matthew Huentelman, Hilleke E Hulshoff Pol, Masashi Ikeda, Clifford R Jack Jr, Mark Jenkinson, Robert Johnson, Erik G Jönsson, J Wouter Jukema, René S Kahn, Ryota Kanai, Iwona Kloszewska, David S Knopman, Peter Kochunov, John B Kwok, Stephen M Lawrie, Hervé Lemaître, Xinmin Liu, Dan L Longo, Oscar L Lopez, Simon Lovestone, Oliver Martinez, Jean-Luc Martinot, Venkata S Mattay, Colm McDonald, Andrew M McIntosh, Francis J McMahon, Katie L McMahon, Patrizia Mecocci, Ingrid Melle, Andreas Meyer-Lindenberg, Sebastian Mohnke, Grant W Montgomery, Derek W Morris, Thomas H Mosley, Thomas W Mühleisen, Bertram Müller-Myhsok, Michael A Nalls, Matthias Nauck, Thomas E Nichols, Wiro J Niessen, Markus M Nöthen, Lars Nyberg, Kazutaka Ohi, Rene L Olvera, Roel A Ophoff, Massimo Pandolfo, Tomas Paus, Zdenka Pausova, Brenda WJH Penninx, G Bruce Pike, Steven G Potkin, Bruce M Psaty, Simone Reppermund, Marcella Rietschel, Joshua L Roffman, Nina Romanczuk-Seiferth, Jerome I Rotter, Mina Ryten, Ralph L Sacco, Perminder S Sachdev, Andrew J Saykin, Reinhold Schmidt, Helena Schmidt, Peter R Schofield, Sigurdur Sigursson, Andy Simmons, Andrew Singleton, Sanjay M Sisodiya, Colin Smith, Jordan W Smoller, Hilkka Soininen, Vidar M Steen, David J Stott, Jessika E Sussmann, Anbupalam Thalamuthu, Arthur W Toga, Bryan Traynor, Juan Troncoso, Magda Tsolaki, Christophe Tzourio, Andre G Uitterlinden, Maria C Valdés Hernández, Dennis Van 't Ent, Marcel Van der Brug, Aad Van der Lugt, Nic JA Van der Wee, Neeltje EM Van Haren, Marie-Jose Van Tol, Badri N Vardarajan, Bruno Vellas, Dick J Veltman, Henry Völzke, Henrik Walter, Joanna M Wardlaw, Thomas H Wassink, Michael E Weale, Daniel R Weinberger, Michael W Weiner, Wei Wen, Eric Westman, Tonya White, Tien Y Wong, Clinton B Wright, Ronald H Zielke, Alan B Zonderman, Nicholas G Martin, Cornelia M Van Duijn, Margaret J Wright, WT Longstreth Jr, Gunter Schumann, Hans J Grabe, Barbara Franke, Lenore J Launer, Sarah E Medland, Sudha Seshadri, Paul M Thompson, M Arfan Ikram
